# Genetic architecture of berry aroma compounds in a QTL (quantitative trait loci) mapping population of interspecific hybrid grapes (*Vitis labruscana* × *Vitis vinifera*)

**DOI:** 10.1186/s12870-022-03842-z

**Published:** 2022-09-23

**Authors:** Kazuya Koyama, Atsushi Kono, Yusuke Ban, Sharon Marie Bahena-Garrido, Tomoko Ohama, Kazuhiro Iwashita, Hisashi Fukuda, Nami Goto-Yamamoto

**Affiliations:** 1 National Research Institute of Brewing, 3–7–1 Kagamiyama, Higashihiroshima, Hiroshima 739–0046 Japan; 2 Institute of Fruit Tree and Tea Science, NARO, 2-1 Fujimoto, Tsukuba, Ibaraki 305-8605 Japan; 3 Western Region Agricultural Research Center (Kinki, Chugoku and Shikoku Regions), NARO, 6-12-1 Nishifukatsu, Fukuyama, Hiroshima 721-8514 Japan

**Keywords:** Volatile compounds, Quantitative trait locus (QTL) analysis, *Vitis labruscana*, *Vitis vinifera*, Secondary metabolism, Terpenoids, Norisoprenoids

## Abstract

**Background:**

Although grapes accumulate diverse groups of volatile compounds, their genetic regulation in different cultivars remains unelucidated. Therefore, this study investigated the volatile composition in the berries of an interspecific hybrid population from a *Vitis labruscana* ‘Campbell Early’ (CE) × *Vitis vinifera* ‘Muscat of Alexandria’ (MA) cross to understand the relationship among volatile compounds and their genetic regulation. Then, a quantitative trait locus (QTL) analysis of its volatile compounds was conducted.

**Results:**

While MA contained higher concentrations of monoterpenes and norisoprenoids, CE contained higher concentrations of C6 compounds, lactones and shikimic acid derivatives, including volatiles characteristic to American hybrids, i.e., methyl anthranilate, *o*-aminoacetophenone and mesifurane. Furthermore, a cluster analysis of volatile profiles in the hybrid population discovered ten coordinately modulated free and bound volatile clusters. QTL analysis identified a major QTL on linkage group (LG) 5 in the MA map for 14 monoterpene concentrations, consistent with a previously reported locus. Additionally, several QTLs detected in the CE map affected the concentrations of specific monoterpenes, such as linalool, citronellol and 1,8-cineol, modifying the monoterpene composition in the berries. As for the concentrations of five norisoprenoids, a major common QTL on LG2 was discovered first in this study. Several QTLs with minor effects were also discovered in various volatile groups, such as lactones, alcohols and shikimic acid derivatives.

**Conclusions:**

An overview of the profiles of aroma compounds and their underlying QTLs in a population of interspecific hybrid grapes in which muscat flavor compounds and many other aroma compounds were mixed variously were elucidated. Coordinate modulation of the volatile clusters in the hybrid population suggested an independent mechanism for controlling the volatiles of each group. Accordingly, specific QTLs with significant effects were observed for terpenoids, norisoprenoids and some volatiles highly contained in CE berries.

**Supplementary Information:**

The online version contains supplementary material available at 10.1186/s12870-022-03842-z.

## Background

*Vitis vinifera* is a grape species originating from western Asia between the Black and Caspian seas. It is widely distributed worldwide and is considered the most commercially important species for producing wine and fresh fruits because of its superior berry quality. However, it is susceptible to many diseases [1, 2]. Contrastively, *Vitis labrusca* originating from North America is resistant to several diseases and low temperature. Therefore, their interspecific hybrid cultivars called *Vitis labruscana*, which inherited the characteristics of *V. labrusca*, are cultivated in North America and Japan to produce fresh fruits, juice and wine [3].

Aroma compounds in berries are essential because these compounds determine the quality of grapes and wines, affecting consumer preference. Studies have reported that many volatile compounds are responsible for the aroma of grapes [4–6]. These chemical groups (Additional file 4: Table S1) include lipid derivatives (C_6_ compounds, lactones, alcohols and aldehydes and esters as subgroups), shikimic acid derivatives (benzenes, volatile phenols and vanillins as subgroups), terpenoids and C_13_ norisoprenoids. Monoterpenes, mainly present as alcohols, contribute to the primary floral aroma of muscat grapes [7]. Some *V. vinifera* cultivars contain many of these compounds that match consumer preferences [3]. However, C_13_ norisoprenoids, derived from carotenoid degradation, also contain many distinct aroma compounds [8]. For example, *V. labrusca* has a distinct aroma profile from *V. vinifera*. While both methyl anthranilate and *o*-aminoacetophenone have been implicated as critical to the perception of the “foxy” aroma of *V. labrusca* and *V. labruscana* [9], furaneol and its methoxylated derivative, mesifurane, are found at concentrations over the threshold, which contributes to the “strawberry” aroma of many *V. labruscana* cultivars [10]. These aroma compounds are contained in low abundance in grapes. However, because of their low odour threshold, they significantly contribute to the sensory characteristics of grapes. A comprehensive volatilome of *V. labruscana* cultivars by headspace solid-phase microextraction-gas chromatography-mass spectrometry (SPME-GCMS) analysis reported their distinct volatile profiles from those of *V. vinifera* [4, 11]. However, since the polar and slightly volatile aroma compounds characteristic of *V. labruscana*, such as methyl anthranilate, were undetected using this analytical method, further studies are needed.

Additionally, many aroma compounds in berries are accumulated in non-volatile and bound forms, which are most commonly the glycosylated precursors. Although free forms determine the aroma of table grapes, all non-volatile precursors serve as potential aroma reservoirs for wine and are converted into various aromas upon hydrolysis by yeast enzymes during fermentation and ageing conditions under low pH [4, 6, 12]. Alternatively, while grape berries usually contain bound volatiles at higher concentrations than free volatiles, the quality and quantity of bound precursors are considered to be free volatile determinants, as suggested in other species [13, 14]. Thus, various studies have tried investigating the genetic regulation of volatiles, primarily on bound volatiles [14–16].

Molecular biology which aims to understand the secondary metabolism of grapes has developed unprecedentedly. Powerful resources, such as the availability of whole-genome sequences [17] and the data accumulation of omics platforms, have also revealed the genes and enzymes related to the aroma regulation in grapes [18–25]. In berries, various monoterpenes are synthesised from isopentenyl diphosphate, a universal building block mainly produced via the mevalonate-independent pathway. Battilana et al. [15] identified a major quantitative trait locus (QTL) in linkage group (LG) 5 for three monoterpenes, using two *V. vinifera* × *V. vinifera* and *V. vinifera* × *V. riparia* grape populations. A subsequent association study revealed that the polymorphisms of the 1-deoxy-D-xylulose-5-phosphate synthase class I gene (*VvDXS1*) of the methyl-erythritol-phosphate (MEP) pathway are responsible for the muscat flavor in grapes [26]. However, for diverse volatile groups except for terpenoids, no similar studies have identified the genes or steps on their metabolic pathways, which determine their varietal differences in grapes. In this context, additional studies delimiting the chromosome regions linked to each volatile group in the berries will help understand the whole picture of the genetic regulation of aromas by interconnecting with existing knowledge on their metabolism.

For this study’s QTL analysis, a bi-parental population (Pop AC) was generated by crossing *V. labruscana* ‘Campbell Early’ (CE) and *V. vinifera* ‘Muscat of Alexandria’ (MA), which are used as table and wine grapes in Japan [27]. This population is considered particularly useful for investigating the genetic regulation of various volatile groups because the individuals show extensive diversity of volatile compounds inherited from both parent cultivars. Therefore, this study investigated the relationship among the volatile compounds in the hybrid population, after which we elucidated their genetic regulation. First, the composition of both free and bound volatile compounds in the berry populations was comprehensively analysed, including the volatiles characteristic of the aroma of *V. labruscana*. Then, a QTL analysis of these volatile compounds was conducted.

## Results

### Differences in the volatile composition

**Fig. 1:**
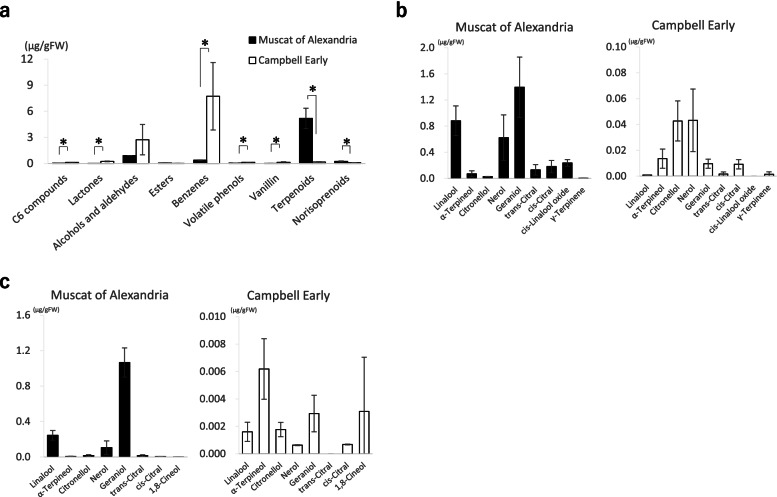
Volatile compositions in ‘Muscat of Alexandria’ and ‘Campbell Early’ berries. Both the bound and free volatiles were classified into nine chemical groups. Then, the total concentrations of each group averaged over 2 years (**a**), and the concentrations of individual bound (**b**) and free (**c**) monoterpene of the two cultivars were compared. Asterisks indicate the significant differences between the cultivars after 2 years at *P* < 0.05 by *t*-test

The volatile composition of berries from MA and CE was analysed. Since these groups represented the potential reserves of aroma compounds in berries, both free and bound volatile groups were analysed. As a result, 29 free and 66 bound volatiles were identified (Additional file 4: Table S1). We also observed that the volatile composition profiles of the two-parent cultivars were significantly different (Fig. 1, Additional file 1: Fig. S1). Specifically (Fig. 1a), while the total terpenoids and norisoprenoids concentrations in MA for 2 years were significantly higher than those in CE, those of C_6_ compounds, lactones and shikimic acid derivatives (benzenes, volatile phenols and vanillins) in CE were significantly higher than those in MA. Thus, the volatiles identified in MA were mostly composed of terpenoids, benzenes, alcohols and aldehydes, while those in CE were mostly composed of benzenes, lactones, alcohols and aldehydes. Results also showed that these cultivars’ free and bound monoterpene compositions varied remarkably (Fig. 1b, c). For instance, in MA, geraniol was the most abundant terpene in both free and bound forms, followed by linalool and nerol. However, in bound volatiles, linalool oxide was relatively high. While geraniol was not the most abundant terpene in CE, α-terpineol, citronellol and nerol were contained at relatively high levels in both free and bound forms. In addition, CE berries had a higher percentage of bound γ-terpinene and free 1,8-cineol than MA berries (Additional file 1: Fig. S1).

**Fig. 2:**
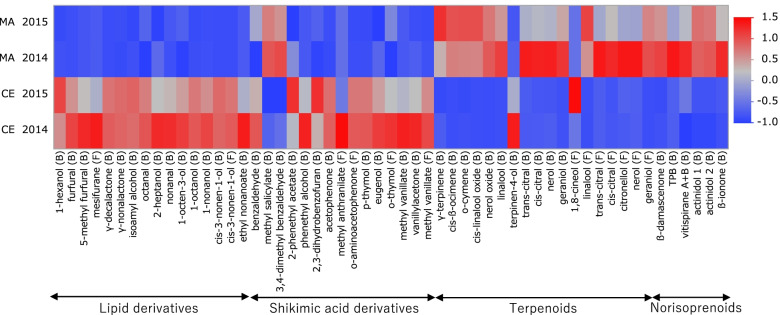
Comparison of the volatile compounds differentially accumulated in the berries of ‘Muscat of Alexandria’ (MA) and ‘Campbell Early’ (CE). Volatile compounds that showed significant differences between the two cultivars were selected (*t*-test, *P* < 0.05). For heatmap visualisation, the concentrations of each compound were normalised based on the average concentrations among all individuals for 2 years. Higher concentrations for each compound are presented in red, whereas lower concentrations are presented in blue, as shown on the scale. The bound volatiles are indicated by (B), whereas the free volatiles are indicated by (F)

Figure 2 shows 55 volatile compounds whose concentrations between cultivars were significantly different. Among lipid derivatives, alcohols, such as bound 1-octanol, 1-nonanol and cis-3-nonen-1-ols, as well as lactones and furans, such as bound γ-decalactone, γ-nonalactone, furfural and free mesifurane, were significantly higher in CE than in MA. For example, mesifurane was specifically contained in CE berries at 187.5 μg/kg. Also, most shikimic acid derivatives in Fig. 2 accumulated higher in CE than in MA. For example, phenethyl alcohol, phenethyl acetate and eugenol accumulated as bound volatiles in CE berries. Additionally, more methyl anthranilate and *o*-aminoacetophenone, “foxy” aroma compounds, were accumulated in CE than in MA. However, since methyl anthranilate and *o*-aminoacetophenone could not be detected in most individuals in Pop AC using stir bar sorptive extraction (SBSE)-GCMS method due to their extremely low concentrations in the berries, these components were analysed by using tandem mass spectrometry (MSMS) with higher sensitivity. Results showed that the concentrations of these compounds in CE berries were 29.9 and 29.8 μg/kg, respectively. In contrast, those of MA were found at extremely low concentrations of 0.3 and 0.9 μg/kg of berries.

On the other hand, higher concentrations of methyl salicylate and 3,4-dimethyl benzaldehyde among shikimic acid derivatives were detected in MA than in CE. Moreover, all monoterpenes and norisoprenoids in Fig. 2 accumulated at higher levels in MA than in CE, except 1,8-cineol, which was reported to contribute to menthol and overall green aromatic expression in red wine [28]. To confirm the absence of interference in the coeluting peaks and increase the reliability of the identification among various monoterpenes with structural similarity, 1,8-cineol was analysed using tandem MS. Results showed that the concentrations of 1, 8-cineol among Pop AC obtained from the two analytical methods were consistent (data not shown).

### Relationships among volatiles

**Fig. 3:**
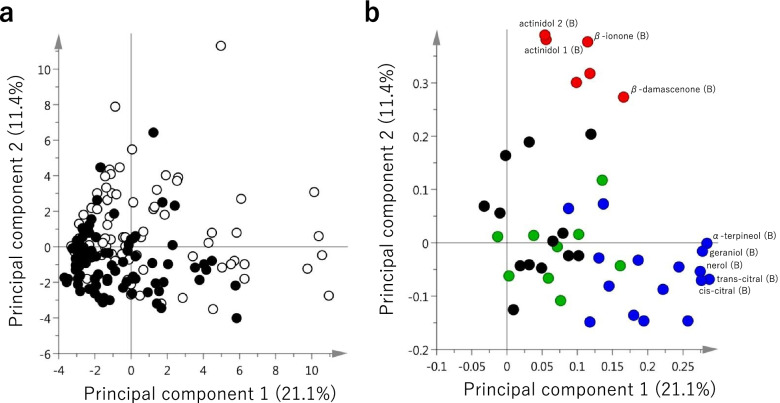
The PCA of the free and bound volatile compounds after 2 years, contained in the F1 hybrid population (Pop AC). **a** The first two-component scores of individuals harvested in 2014 (open circles) and 2015 (closed circles). **b** Corresponding loading plot of the variables, showing the positions of the volatiles classified as lipid derivatives (green), shikimic acid derivatives (black), terpenoids (blue) and norisoprenoids (red). Selected variables to interpret the score plot are labelled on the loading plot

The profiles of volatile compounds contained in Pop AC berries were analysed for 2 years. First, the 45 major bound and free volatiles from all chemical groups were quantified. Subsequently, principal component analysis (PCA) was conducted to analyse Pop AC’s variability in volatile compositions (Fig. 3). The principal components 1 (PC1) and 2 (PC2) contributed 21.1 and 11.4% of the total variance, respectively. We also observed that while PC1 was positively correlated with several monoterpenes, such as trans- and cis-citral and α-terpineol, PC2 was positively correlated with norisoprenoids, such as actinidol and β-ionone. However, the distributions of individuals in 2014 and 2015 on the score plot were well-overlapped. We also observed that the concentrations of the volatiles showed a similar tendency within 2 years, and 27 of 45 volatiles showed a coefficient of determination higher than 0.35 (Additional file 2: Fig. S2). Differences in climate conditions between the 2 years (Additional file 4: Table S2) or other factors may affect the variabilities between vintages, since the volatiles, such as terpenoids and norisoprenoids, are known to be affected by the environment [6, 23, 29, 30].

**Fig. 4:**
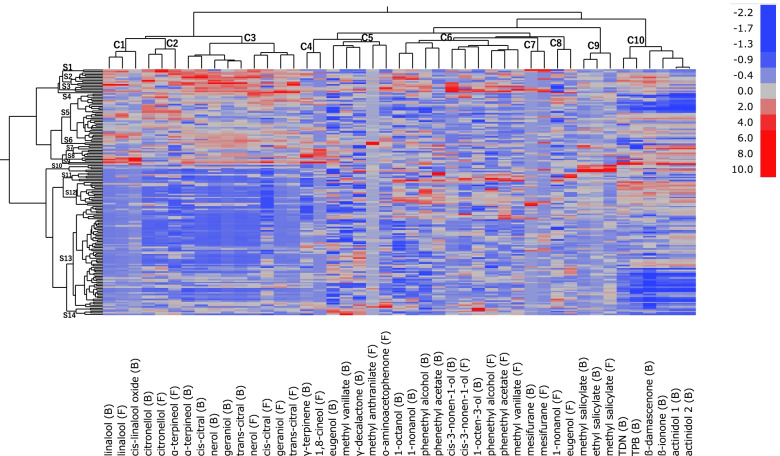
Heatmap with two-dimensional hierarchical dendrograms of the volatiles in the hybrid population (Pop AC). Each column in the heat map represents a volatile, and each row represents a genotype. The upper dendrogram corresponds to the volatiles, after which the clusters are indicated by C1–C10. The left dendrogram corresponds to the individuals, indicating the clusters by S1–S14. For heatmap visualisation, the concentrations of each compound were normalised based on the average concentration among all individuals for 2 years. Higher concentrations for each compound are presented in red, whereas lower concentrations are presented in blue, as shown on the scale on the right. Bound volatiles are indicated by (B), whereas the free volatiles are indicated by (F)

Furthermore, to analyse the relationships among the volatiles, hierarchical clustering and correlation matrix analyses were performed on the volatile data of both years (Fig. 4, Additional file 5: Table S3, Additional file 6: Table S4). Cluster analysis grouped the volatile compounds into ten main clusters (C1–C10). The compounds with the same chemical group or similar chemical nature were generally grouped into the same or close clusters. Results also showed that same compounds’ free and bound forms showed a close relationship (Additional file 5: Table S3, Additional file 6: Table S4), as suggested in previous studies [13, 14]. As shown, C1–C3 were composed of many terpenoids which were contained higher in MA than in CE. While C1 was composed of linalool and its derivatives, C2 was composed of citronellol and α-terpineol (free), and C3 was composed of nerol, geraniol, trans- and cis-citral. On the other hand, γ-terpinene and 1,8-cineol, which were contained at a higher percentage in CE, were grouped in another cluster (C4). C5 comprised shikimic acid derivatives and lactones which are highly contained in CE, such as eugenol, methyl vanillate and γ-decalactone, as well as methyl anthranilate and *o*-aminoacetophenone in a sub-cluster. Also, C6 comprised alcohols and shikimic acid derivatives highly contained in CE compared to MA. For example, bound 1-octanol, 1-nonanol, cis-3-nonen-1-ol, 1-octen-3-ol and phenethyl alcohol were included in the same cluster (C6). Moreover, phenethyl alcohol and its acetic ester, phenethyl acetate, were closely correlated, suggesting the former concentration as the limiting factor for the latter. Mesifurane was grouped in another cluster (C7), while methyl salicylate and ethyl salicylate were grouped in C9. All norisoprenoids identified were grouped in C10.

Based on individual genotypes, the cluster analysis grouped them into 14 main clusters (S1–S14). Genotypes S1–S9 contained higher concentrations of terpenoids than the other genotype clusters, and specific monoterpenes were abundant in each cluster. Genotypes S1–S4 and S11–S12 contained high concentrations of some alcohols and shikimic acid derivatives in C6. Results also showed that genotypes S1–S4, S7–S9 and S12 contained higher concentrations of almost all the norisoprenoids than the other genotype clusters, which is consistent with the high correlation coefficients among the norisoprenoid concentrations (Additional file 5: Table S3, Additional file 6: Table S4).

Summarily, while volatiles in the same chemical groups were generally classified in the same or close clusters, each genotype cluster accumulated different combinations of volatile clusters.

### QTL analysis of the volatile compounds

**Fig. 5:**
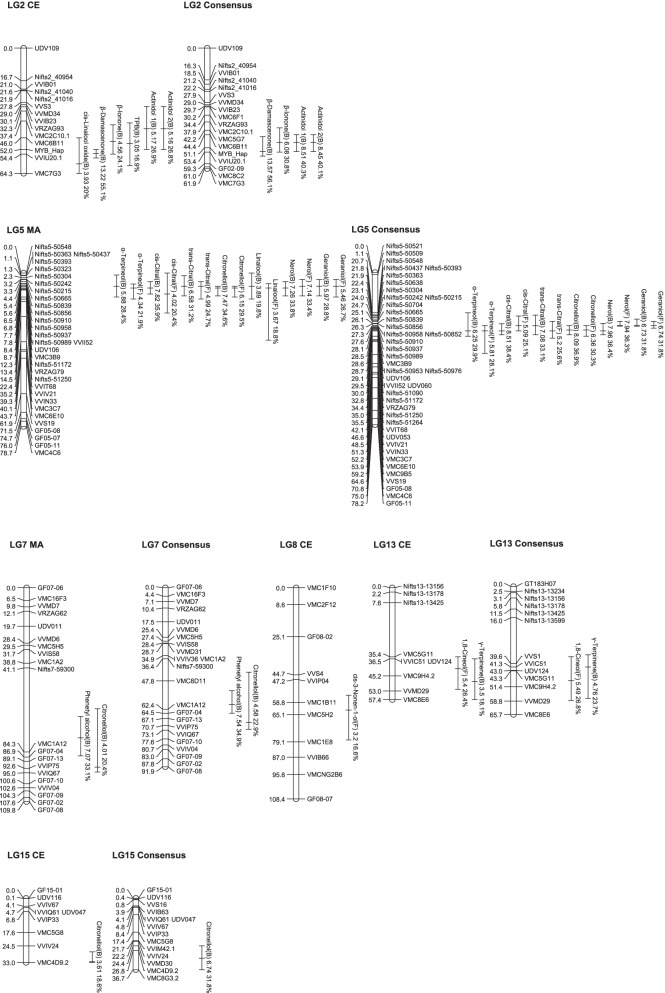
Locations of consistent volatile QTLs identified during both years in the hybrid population (Pop AC). QTLs obtained from the analysis with the average data of 2 years are shown. A vertical line on the right of linkage map indicates the position of LOD peak (middle bar) and one LOD confidence interval (between top and bottom bars) of each QTL. The volatile name is followed by the LOD peak and the percentage variance explained by the QTL. Distances of markers from the top of each linkage map are indicated in cM

The frequency distribution of the concentrations of each volatile was comparable for 2 years and showed a continuous variation, which is typical for quantitative traits (Additional file 2: Fig. S2). This study observed transgressive segregation in the population in all volatiles. Then, QTL analysis was performed using the dataset of the volatile composition for each year (2014 and 2015) and the mean values over 2 years. They observed that many QTLs were detected for volatile production, with a LOD more than the threshold of α = 0.05 (Additional file 4: Table S5). Consistent QTLs, which were detected in both years, are shown in Table 1, and the positions of their LOD peaks and one LOD confidence intervals which were obtained from the analysis with the data averaged over 2 years were visually presented in Fig. 5.

**Table 1:** Consistent QTLs of the volatile concentrations identified during both years in the hybrid population (Pop AC)

Volatile^**a**^	Chemical group	Year	***n***^**b**^	Linkage group (LG)	Map^**c**^	LOD peak	LOD threshold α = 0.05	% variance explained	QTL position (cM)	Confidence interval 1.0-LOD (cM)	Nearest marker^**d**^
cis-3-Nonen-1-ol (F)	Alcohols and aldehydes	2014	82	8	CE	2.98	2.8	15.2	70.1	54.2–97.8	VMC5H2
		2015	90	8	CE	2.91	2.6	14	62.8	56.2–76.1	VMC5H2
		Mean	81	8	CE	3.2	2.7	16.6	62.8	55.2–78.1	VMC5H2
Phenetyl alcohol (B)	Benzenes	2014	82	7	MA	5.8	3	27.8	80.1	66.1–86.9	VMC1A12
		2015	90	7	MA	6.14	2.9	27	67.1	55.1–80.1	VMC1A12
		Mean	81	7	MA	7.07	2.9	33.1	77.1	66.1–86.3	VMC1A12
		2014	82	7	Consensus	6.77	4.5	31.6	61.8	53.8–64.4	VMC1A12
		2015	90	7	Consensus	6.48	4.2	28.2	50.8	44.4–59.8	VMC8D11
		Mean	81	7	Consensus	7.54	4.5	34.9	59.8	52.8–64.4	VMC1A12
1,8-Cineol (F)	Terpenoids	2014	82	13	CE	3.86	2.5	19.3	48.2	32.6–56.0	VMC9H4.2
		2015	90	13	CE	5.88	2.6	26.2	41.5	34.6–49.2	VMC9H4.2
		Mean	81	13	CE	5.4	2.5	26.4	41.5	35.4–52.2	VMC9H4.2
		2015	90	13	Consensus	5.86	4.9	26.1	48.3	35.0–56.4	VMC9H4.2
		Mean	81	13	Consensus	5.49	5	26.8	48.3	36.0–58.4	VMC9H4.2
*γ*-Terpinene (B)	Terpenoids	2014	82	13	CE	2.78	2.5	14.5	48.2	30.6–57.4	VMC9H4.2
		2015	90	13	CE	4.42	2.6	20.2	48.2	38.5–56.0	VMC9H4.2
		Mean	81	13	CE	3.5	2.5	18.1	48.2	36.5-Bottom	VMC9H4.2
		2014	82	13	Consensus	5.28	4.8	25.7	41.3	36.0–47.3	VVIC51
		Mean	81	13	Consensus	4.76	4.7	23.7	41.3	35.0–57.4	VVIC51
cis-Linalool oxide (B)	Terpenoids	2014	82	2^e^	CE	3.57	2.5	18.2	59.4	43.4-Bottom	VMC7G3
		2015	90	2^e^	CE	2.51	2.3	12.1	57.4	42.4-Bottom	VVIU20.1
		Mean	81	2^e^	CE	3.93	2.6	20	59.4	46.0-Bottom	VVIU20.1
*α*-Terpineol (B)	Terpenoids	2014	82	5^f^	MA	3.65	2.8	18.5	6.8	0.0–18.5	Nifts5–50958
		2015	90	5^f^	MA	9.42	2.7	38.3	2.3	0.0–12.3	Nifts5–50304
		Mean	81	5^f^	MA	5.88	2.9	28.4	6.8	1–12.3	Nifts5–50958
		2015	90	5^f^	Consensus	9.98	4.5	40	23.1	0.0–24.7	Nifts5–50304
		Mean	81	5^f^	Consensus	6.25	4.5	29.9	30	20.7–32.8	Nifts5–51090
*α*-Terpineol (F)	Terpenoids	2014	82	5^f^	MA	3.44	2.8	17.4	7.7	5.6–13.3	Nifts5–50937
		2015	90	5^f^	MA	4.51	2.7	20.8	7.7	5.4–13.4	Nifts5–50937
		Mean	81	5^f^	MA	4.34	2.9	21.9	7.7	5.6–13.3	Nifts5–50937
		2015	90	5^f^	Consensus	6.1	4.5	27.1	30	27.3–41.5	Nifts5–51090
		Mean	81	5^f^	Consensus	5.81	4.6	28.1	30	27.3–41.5	Nifts5–51090
cis-Citral (B)	Terpenoids	2014	82	5^f^	MA	6.66	2.8	31.2	7.7	4.4–11.7	Nifts5–50937
		2015	90	5^f^	MA	9.05	2.9	37.1	7.7	3.3–12.3	Nifts5–50937
		Mean	81	5^f^	MA	7.82	2.7	35.9	7.7	4.4–10.7	Nifts5–50937
		2014	82	5^f^	Consensus	7.59	4.6	34.7	30	26.3–32.0	Nifts5–51090
		2015	90	5^f^	Consensus	9.36	4.6	38.1	27.3	24.0–32.8	Nifts5–50852
		Mean	81	5^f^	Consensus	8.51	4.8	38.4	30	26.3–32.0	Nifts5–51090
cis-Citral (F)	Terpenoids	2014	82	5^f^	MA	3.41	2.8	17.2	4.4	0.0–13.3	Nifts5–50665
		2015	90	5^f^	MA	3.08	2.6	14.7	10.7	3.3–20.5	Nifts5–51172
		Mean	81	5^f^	MA	4.02	2.8	20.4	10.7	0.0–20.5	Nifts5–51172
		Mean	81	5^f^	Consensus	5.09	4.6	25.1	27.3	22.4–30.0	Nifts5–50852
trans-Citral (B)	Terpenoids	2014	82	5^f^	MA	4.77	2.8	23.5	6.8	0.0–12.3	Nifts5–50958
		2015	90	5^f^	MA	9.72	2.8	39.2	6.8	3.3–11.7	Nifts5–50958
		Mean	81	5^f^	MA	6.58	2.8	31.2	6.8	1.3–11.7	Nifts5–50958
		2014	82	5^f^	Consensus	5.22	4.6	25.4	30	20.7–35.0	Nifts5–51090
		2015	90	5^f^	Consensus	11.14	4.5	43.5	27.3	26.3–27.6	Nifts5–50852
		Mean	81	5^f^	Consensus	7.08	4.6	33.1	27.3	21.9–32.0	Nifts5–50852
trans-Citral (F)	Terpenoids	2014	82	5^f^	MA	3.7	2.8	18.6	5.4	4.4–13.3	Nifts5–50958
		2015	90	5^f^	MA	3.7	2	17.4	6.8	0.0–21.5	Nifts5–50958
		Mean	81	5^f^	MA	4.99	2.8	24.7	6.8	4.4–13.3	Nifts5–50958
		2015	90	5^f^	Consensus	4.63	4.6	21.3	27.3	21.9–32.8	Nifts5–50958
		Mean	81	5^f^	Consensus	5.2	4.5	25.6	30	25.1–33.8	Nifts5–51090
Citronellol (B)	Terpenoids	2014	82	5^f^	MA	7.15	2.8	33.1	7.7	5.6–12.3	Nifts5–50937
		2014	82	7	MA	3.11	2.8	16	92.1	60.1–92.1	VVIP75
		2015	90	5^f^	MA	6.8	2.7	29.4	7.7	5.6–12.3	Nifts5–50937
		2015	90	7	MA	2.88	2.7	13.7	92.6	54.1–106.3	VVIP75
		Mean	81	5^f^	MA	7.47	2.7	34.6	7.7	6.8–11.7	Nifts5–50937
		Mean	81	7	MA	4.01	2.7	20.4	92.1	62.1–94.6	VVIP75
		2014	82	15	CE	3.26	2.8	16.7	31.5	26.5–33.0	VMC4D9.2
		2015	90	15	CE	3.76	2.8	17.5	33	29.5-Bottom	VMC4D9.2
		Mean	81	15	CE	3.61	2.8	18.6	32.5	27.5-Bottom	VMC4D9.2
		2014	82	5^f^	Consensus	7.86	4.5	35.7	29.5	27.3–32.0	VVII52
		2014	82	15	Consensus	5.85	4.5	28	31.8	24.4-Bottom	VMC8G3.2
		2015	90	5^f^	Consensus	7.27	4.9	31.1	29.5	27.3–32.8	VVII52
		2015	90	15	Consensus	6.91	4.9	29.8	29.8	25.4-Bottom	VMC4D9.2
		Mean	81	5^f^	Consensus	8.09	4.7	36.9	29.5	27.3–32.0	VVII52
		Mean	81	7	Consensus	4.58	4.7	22.9	69.1	43.4–72.7	VVIP75
		Mean	81	15	Consensus	6.74	4.7	31.8	30.8	24.4-Bottom	VMC4D9.2
Citronellol (F)	Terpenoids	2014	82	5^f^	MA	6.02	2.7	28.4	7.7	6.8–11.7	Nifts5–50937
		2015	90	5^f^	MA	5.32	2.2	24.1	7.7	6.8–14.4	Nifts5–50937
		Mean	81	5^f^	MA	6.15	2.4	29.5	7.7	6.8–12.3	Nifts5–50937
		2014	82	5^f^	Consensus	6.08	4.7	28.6	28.1	27.3–32.8	Nifts5–50937
		2015	90	5^f^	Consensus	5.83	4.6	26	29.5	27.3–32.8	VVII52
		Mean	81	5^f^	Consensus	6.36	4.6	30.3	29.5	27.3–32.8	VVII52
Linalool (B)	Terpenoids	2014	82	5^f^	MA	3.05	2.4	15.7	4.3	0.0–19.5	Nifts5–50665
		2015	90	5^f^	MA	5.24	2.3	23.5	2.3	0.0–5.4	Nifts5–50304
		Mean	81	5^f^	MA	3.89	2.3	19.8	6.8	0.0–13.3	Nifts5–50958
		2015	90	5^f^	Consensus	5.61	4.7	24.9	30	26.1–32.8	Nifts5–51090
Linalool (F)	Terpenoids	2014	82	5^f^	MA	3.33	2.5	16.9	7.7	5.4–21.5	Nifts5–50937
		2015	90	5^f^	MA	3.47	2.6	16.4	6.8	5.4–36.2	Nifts5–50958
		Mean	81	5^f^	MA	3.67	2.8	18.8	7.7	5.4–34.4	Nifts5–50937
Nerol (B)	Terpenoids	2014	82	5^f^	MA	4.32	2.8	21.6	7.7	3.3–13.3	Nifts5–50937
		2015	90	5^f^	MA	12.55	2.9	47.4	7.7	5.6–11.7	Nifts5–50937
		Mean	81	5^f^	MA	7.26	3.1	33.8	7.7	4.4–11.7	Nifts5–50937
		2014	82	5^f^	Consensus	4.95	4.7	24.3	30	24.0–32.8	Nifts5–51090
		2015	90	5^f^	Consensus	12.8	4.4	48.1	30	28.6–32.0	Nifts5–51090
		Mean	81	5^f^	Consensus	7.96	4.6	36.4	30	26.1–32.0	Nifts5–51090
Nerol (F)	Terpenoids	2014	82	5^f^	MA	6.82	2.8	31.5	6.8	1.3–11.7	Nifts5–50958
		2015	90	5^f^	MA	7.45	2.7	32	7.7	4.4–13.3	Nifts5–50937
		Mean	81	5^f^	MA	7.14	2.8	33.4	6.8	3.3–11.7	Nifts5–50958
		2014	82	5^f^	Consensus	7.53	4.7	34.2	27.3	24.0–27.6	Nifts5–50958
		2015	90	5^f^	Consensus	8.07	4.8	34.1	27.3	25.1–32.0	Nifts5–50852
		Mean	81	5^f^	Consensus	7.94	4.6	36.3	27.3	25.1–27.6	Nifts5–50852
Geraniol (B)	Terpenoids	2014	82	5^f^	MA	3.86	2.7	19.5	6.8	3.3–12.3	Nifts5–50958
		2015	90	5^f^	MA	10.07	2.9	40.3	2.3	0.0–11.7	Nifts5–50304
		Mean	81	5^f^	MA	5.97	2.9	28.8	6.8	3.3–11.7	Nifts5–50958
		2015	90	5^f^	Consensus	11.69	4.5	45	27.3	26.3–27.6	Nifts5–50852
		Mean	81	5^f^	Consensus	6.73	4.5	31.8	27.3	24.0–27.6	Nifts5–50958
Geraniol (F)	Terpenoids	2014	82	5^f^	MA	5.42	2.7	26	6.8	0.0–11.7	Nifts5–50958
		2015	90	5^f^	MA	4.25	2.1	19.7	6.8	3.3–20.5	Nifts5–50958
		Mean	81	5^f^	MA	5.46	2.6	26.7	6.8	3.3–12.3	Nifts5–50958
		2014	82	5^f^	Consensus	7.09	4.3	32.5	27.3	25.1–28.6	Nifts5–50958
		2015	90	5^f^	Consensus	4.93	4.9	22.5	27.3	25.1–32.8	Nifts5–50958
		Mean	81	5^f^	Consensus	6.74	4.9	31.8	27.3	25.1–28.6	Nifts5–50958
*ß*-Damascenone (B)	Norisoprenoids	2014	82	2	CE	8.43	2.8	39.6	54	47.0–57.4	VVIU20.1
		2015	90	2	CE	13.57	2.8	50.1	53	47.0–56.4	MYB-Hap
		Mean	81	2	CE	13.22	2.8	55.1	54	52.0–56.4	VVIU20.1
		2014	82	2	Consensus	9.03	4.3	41.7	48.4	44.2–55.4	MYB-Hap
		2015	90	2	Consensus	13.78	4.5	50.6	52.1	46.4–55.4	MYB-Hap
		Mean	81	2	Consensus	13.57	4.3	56.1	53.1	45.4–55.4	VVIU20.1
*ß*-Ionone (B)	Norisoprenoids	2014	82	2	CE	2.93	2.5	16.1	48	46.0–60.4	VMC6B11
		2015	90	2	CE	5.12	2.4	23.1	42.4	34.3–54.0	VMC6B11
		Mean	81	2	CE	4.56	2.6	24.1	48	39.4–54.4	VMC6B11
		2015	90	2	Consensus	5.52	4.5	24.6	48.4	37.9–53.4	MYB-Hap
		Mean	81	2	Consensus	6.08	4.6	30.8	48.4	40.9–53.4	MYB-Hap
TPB (B)	Norisoprenoids	2015	90	2	CE	6.17	2.9	27.1	41.4	34.3–46.0	VMC2C10.1
		Mean	81	2	CE	3.05	2.8	16.9	49	38.4–60.4	MYB-Hap
		2015	90	2	Consensus	7.71	4.5	32.6	49.4	44.4–53.1	MYB-Hap
Actinidol 1 (B)	Norisoprenoids	2014	82	2	CE	4.35	2.8	22.9	42.4	29.0–54.0	VMC6B11
		2015	90	2	CE	4.68	2.5	21.3	41.4	32.3–52.0	VMC2C10.1
		Mean	81	2	CE	5.17	2.9	26.9	41.4	30.0–52.0	VMC2C10.1
		2014	82	2	Consensus	6.97	4.6	34.1	48.4	40.9–53.1	MYB-Hap
		2015	90	2	Consensus	5.21	4.7	23.4	41.9	35.4–56.4	VMC5G7
		Mean	81	2	Consensus	8.51	4.4	40.3	48.4	44.4–53.1	MYB-Hap
Actinidol 2 (B)	Norisoprenoids	2014	82	2	CE	4.38	2.9	23	41.4	29.0–53.0	VMC2C10.1
		2015	90	2	CE	4.92	2.7	22.2	41.4	32.3–52.0	VMC2C10.1
		Mean	81	2	CE	5.16	2.9	26.8	41.4	30.0–52.0	VMC2C10.1
		2014	82	2	Consensus	6.97	4.6	34.1	48.4	38.9–52.1	MYB-Hap
		2015	90	2	Consensus	5.58	4.6	24.8	41.9	35.4–56.4	VMC5G7
		Mean	81	2	Consensus	8.45	4.6	40.1	48.4	44.4–53.1	MYB-Hap

^a^ Bound volatiles are indicated by (B), whereas free volatiles are indicated by (F). ^b^ Number of individuals used for QTL analysis. ^c^ MA: ‘Muscat of Alexandria’, CE: ‘Campbell Early’ ^d^ Marker closest to the position of the LOD peak. ^e^ This QTL on LG2 might be equivalent to the QTL by Battilana et al. [15] and Duchêne et al. [16]. ^f^ This QTL on LG5 might be equivalent to the QTL by Battilana et al. [15] and Duchêne et al. [16]. The full list showing all detected QTLs, which include those only identified in a single year are found in Additional file 4: Table S5

Major QTLs located at the top LG5 of MA and consensus maps were found for 14 monoterpenes in C1–C4, both in the free and bound forms. This QTL explained 14.7–48.1% of the total phenotypic variance. Although the positions of the LOD peaks varied depending on the map, year and terpene compound, they were always located at the top LG5, and the confidence intervals based on one LOD of these QTLs were closely overlapped. Subsequently, the candidate genes underlying this QTL in the 12 × PN40024 sequence [17] were searched in the NCBI Map Viewer public database. Investigations revealed that *VvDXS1* (VIT_05s0020g02130, chr5:3851143..3856263) was located near the simple sequence repeats (SSR) markers detected (Nifts5–50937, chr5::3990633..3990829; Nifts5–50958, chr5:4080790..4081000). Since the QTL was detected in MA, which contained abundant terpene compounds, it was thought to increase the concentrations of almost all monoterpenes examined. On the other hand, a minor QTL at the end of LG2 in the CE and consensus maps was found for cis-linalool oxide. This minor QTL was also detected for linalool in the mean of 2 years (Additional file 4: Table S5). Furthermore, two consistent QTLs for bound citronellol were detected in both years on LG7 in the MA map and LG15 in the CE map. These QTLs on LG7 and LG15 explained 13.7–20.4% and 16.7–31.8% of the phenotypic variations, respectively. Therefore, since the same QTLs were not found in other monoterpenes, they may affect particularly the concentrations of citronellol, modifying the monoterpene composition.

Another common QTL for the monoterpenes in C4 was consistently detected on LG13 in the CE and consensus maps for both years. This QTL explained 19.3–26.8% of the phenotypic variations of 1,8-cineol and 14.5–25.7% of γ-terpinene.

However, for the compounds in C6, which belong to alcohols and shikimic acid derivatives, only minor QTLs were detected on LG8 for free cis-3-nonen-1-ol in the CE map and on LG7 for bound phenethyl alcohol in the MA and consensus maps. This QTL on LG8 was also found in other alcohols, such as bound 1-octanol and bound cis-3-nonen-1-ol in 2015, and the common mechanism to control these alcohols were suggested as they were supposed to share a common biosynthetic pathway [21]. A QTL for *o*-aminoacetophenone in C5, which is a foxy aroma compound, was detected in 2014 on LG3 in the CE and consensus maps (16.5–28.3% of explained variance) (Additional file 4: Table S5). Additionally, a QTL for free mesifurane in C7 was detected in 2015 at the top LG12 in the CE and consensus maps (20.6–25.4% of explained variance). The same QTL was found for bound mesifurane in 2014. Since this QTL was detected in CE, which contained high concentrations of free and bound mesifurane, it was thought to increase their concentrations in the berries.

In five of six norisoprenoids in C10, a common QTL was detected on LG2 in the CE and consensus maps for 2 years. Although the LOD peak of β-damascenone was located closer to the end of LG2 than the other norisoprenoids, confidence intervals based on one LOD of these QTLs were well-overlapped. Moreover, the QTL explained 39.6–56.1% of the phenotypic variations in β-damascenone, 16.1–30.8% in β-ionone, 16.9–32.6% in TPB, 21.3–40.3% in actinidol 1 and 22.2–40.1% in actinidol 2. Thus, this QTL had the highest contribution to phenotypic variation in the β-damascenone concentrations of all norisoprenoids. Additionally, since the QTL was detected in CE, which contains lower concentrations of bound norisoprenoids in the berries than MA, this QTL was considered to decrease the concentrations of all norisoprenoids.

### The effect of QTL on the volatile concentrations

**Fig. 6:**
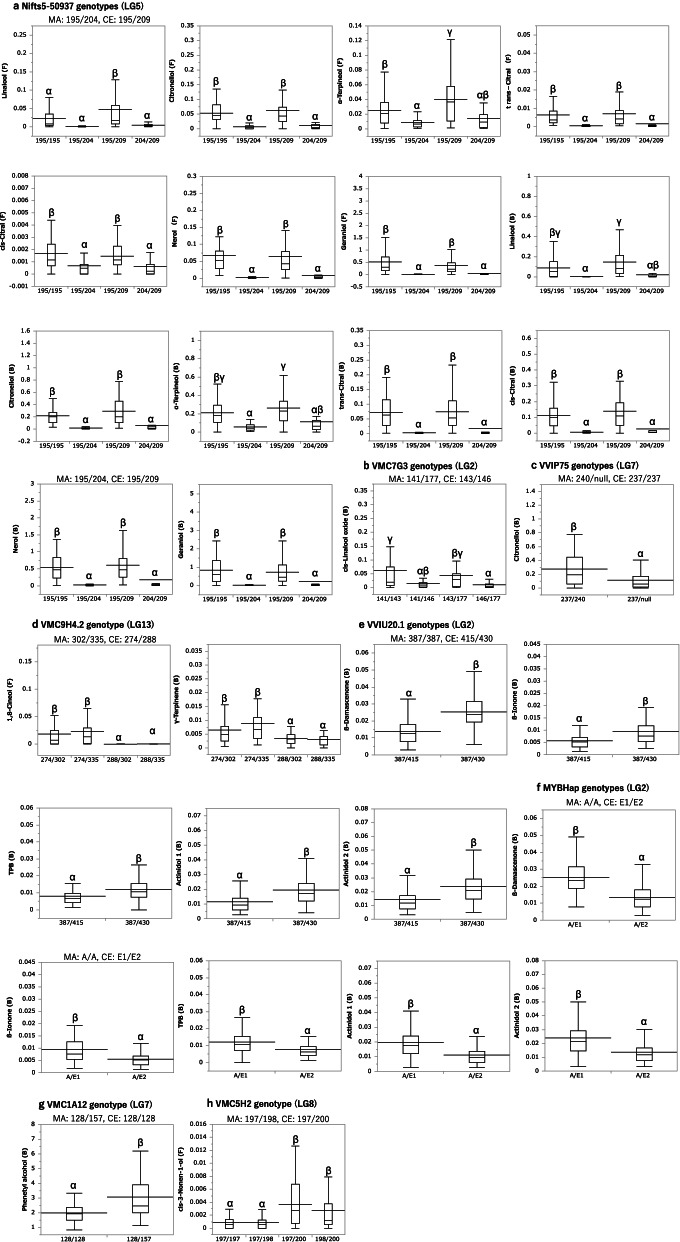
Box plot showing the volatiles concentrations for the genotypes of SSR markers and MYB haplotype nearest to the QTL in the hybrid population (Pop AC). The genotypes of SSR markers are expressed as allele lengths in bp. While the horizontal line inside boxes shows median values, horizontal lines through boxes indicate means. Box height indicate 50% of the data. Different letters (α, β, γ) indicate that the values are significantly different at *P* < 0.05 by Tukey’s HSD. MA: ‘Muscat of Alexandria’, CE: ‘Campbell Early’. The box plot for the genotypes of all SSR markers and MYB haplotype detected are shown in Additional File 3: Fig. S3

We investigated the relationship between the concentrations of the volatiles and the genotypes of the closest markers for each QTL to compare the effect of the QTLs detected in the berries of Pop AC (Fig. 6; Additional file 3: Fig. S3). After several markers had been observed to be closely located to a QTL, common markers among the volatiles in the same chemical groups with significant and consistent effects were selected. Figure 6a shows that the concentrations of seven monoterpenes of both free and bound forms in the progenies with the 195–base pair (bp) allele of Nifts5–50937 on LG5 inherited from MA were significantly higher than the other allele from MA. As for the minor QTL detected for specific monoterpene concentrations, the concentrations of cis-linalool oxide in the progenies with the 146–bpVMC7G3 allele on LG2 inherited from CE were significantly lower than that of the other allele from CE (Fig. 6b). Furthermore, the significant differences in citronellol concentrations occurred among the VVIP75 genotypes on LG7 (Fig. 6c). Figure 6d shows that the concentrations of both 1,8-cineol and γ-terpinene in C4 were significantly higher in the progenies with 274–bp allele of VMC9H4.2 on LG13 inherited from CE than the other allele. However, for five norisoprenoids, progenies with 430–bp allele of VVIU20.1 (Fig. 6e) and Hap E1 of MYB haplotypes on LG2 (Fig. 6f) inherited from CE showed significantly higher concentrations. Finally, for the volatiles in the other chemical groups, such as lactones, alcohols and shikimic acid derivatives, the effects of QTL alleles on the modification of each volatile concentrations in the berries were confirmed by comparing the mean values of the marker genotypes (Fig. 6g, h; Additional File 3: Fig. S3). Thus, these results verified the effects of the QTLs detected for each volatile group in Pop AC.

## Discussion

This comprehensive volatile analysis and clustering revealed that the accumulation of biochemically similar volatiles was modulated similarly among Pop AC, suggesting that their accumulations were under specific genetic control. In fact, QTL analysis found specific QTLs for each group of volatiles as follows:

### Genetic loci related to monoterpene concentrations and composition

In this study, monoterpene compositions were examined in detail, after which ten monoterpene compounds were subjected to QTL analysis, both in free and bound forms. Although a slight influence of vintage on monoterpene concentrations was observed (Additional File 2: Fig. S2), several consistent QTLs obtained for both years suggested that these volatiles were under significant genetic control (Table 1; Fig. 5). It has been reported previously that a key gene, *VvDXS1* was located at the major QTL at the top LG5 [15, 16] and the same locus was found in this study. Similarly, several minor QTLs considered affecting the different monoterpene concentrations were detected (Table 1; Fig. 5), suggesting their contribution to hybrid populations’ wide monoterpene composition varieties. Among them, consistent QTL obtained for both years on LG2 was proposed as equivalent to the previously reported one [15, 16, 31] for linalool. In this study, this locus was also related to the linalool derivative concentrations, i.e., cis-linalool oxide. Hence, considering that the percentages of cis-linalool oxide and linalool in CE were remarkably lower than in MA (Fig. 1b, c; Additional file 1: Fig. S1b, c)), this locus may specifically affect the metabolism of these terpenes, modulating monoterpene composition. Consistent with these results, the effect of this QTL inherited from CE was confirmed to modify the specific monoterpenes (Fig. 6b). Other studies found this QTL in the parent map of aromatic varieties, such as *V. vinifera* ‘Muscat Hamburg’ and *V. vinifera* ‘Muscat Ottonel’ [16, 31]. Therefore, since CE (*V. labrusca* ‘Moore Early’ × (*V. labrusca* ‘Belvidere’ × *V. vinifera* ‘Muscat Hamburg’)) [32] has ‘Muscat of Hamburg’ in its pedigree, it could also inherit this allelic variation on LG2 from this ancestor. Another consistent QTL detected in both years on LG15 in the CE map was specifically related to the regulation of citronellol concentrations, consistent with the remarkably high percentage of citronellol among monoterpenes in CE (Table 1; Figs. 1b, c and 5). However, since previous reports did not focus on this monoterpene, the QTL found here was not reported previously [15, 16, 18, 31, 33].

γ-Terpinene and 1,8-cineol in C4 (Fig. 4) are cyclic monoterpenes with similar structures, and they showed different profiles among the population compared with the other monoterpenes in our study, suggesting a different regulation. Accordingly, a new consistent QTL on LG13 was identified in 2 years for these two terpenes, after which the allele effect on VMC9H4.2 inherited from CE was confirmed to modify these concentrations in the berries (Table 1; Figs. 5 and 6e). For their synthesis, terpene synthases of the TPS-b subfamily [19] were responsible and nine out of 20 TPS-b subfamily genes were localised on LG13 and LG13 random scaffolds in close proximity in the 12 × PN40024 sequence. However, further investigation is necessary to screen for candidate genes within the QTL, including these terpene synthase genes.

### Common genetic loci related to the norisoprenoid concentrations

In berries, only bound norisoprenoids, not the free forms, were detected. Therefore, although a limited contribution of these compounds to the aroma of table grapes was suggested, the bound norisoprenoids detected in this study were considered significantly contributing to the sensory properties of wine [5, 8, 30]. Norisoprenoids are biosynthesised from the oxidative breakdown of carotenoids by 9,10, (9′,10′) carotenoid cleavage dioxygenases. Like monoterpenes, carotenoids can be synthesised in plastids through the MEP pathway. Additionally, they share DXS as a rate-limiting enzyme in their biosynthetic pathways. However, since a correlative relationship between the concentrations of monoterpenes and norisoprenoids was not observed among Pop AC (Fig. 4), another critical step in the norisoprenoid biosynthesis was suggested. Consistent with this hypothesis, we discovered a major consistent QTL detected in both years on LG2, specific for all norisoprenoids identified in this study. Significant differences in norisoprenoid concentrations among the genotypes of VVIU20.1 and MYBHap were also observed (Table 1; Figs. 5 and 6e, f). A previous study reported genes and enzymes putatively involved in the carotenoid metabolic pathway in *V. vinifera* [20]. Particularly, *VvCCD4a* (VIT_02s0087g00910), and *VvCCD4b* (VIT_02s0087g00930) on LG2 are considered to be key genes, accounting for the regulation of norisoprenoids, from their correlative expression patterns to the norisoprenoid accumulation, and their functions, characterised as 9,10, (9′,10′) carotenoid cleavage dioxygenases [18, 23, 25, 34]. However, further investigation is necessary to discover whether these genes or other candidates are positioned within the QTL and to determine their relations to norisoprenoid concentrations in the berries.

### Genetic loci related to the volatile groups contained highly in CE berries

Significantly high volatiles in CE berries, such as lactones alcohols, and shikimic acid derivatives, were modulated differently in Pop AC from the terpenoids and norisoprenoids, which were abundant in MA, suggesting a different regulation of these volatiles (Fig. 4). Among them, phenethyl alcohol was contained at the highest average concentrations in Pop AC (2576 μg/kg berries for bound forms; 1506 μg/kg berries for free forms). These results are consistent with a previous report in which phenethyl alcohol was highly accumulated in non-muscat grape cultivars [35]. Since the concentrations of free phenethyl alcohol were beyond the sensory thresholds reported (1100 μg/kg berries), the compound was considered to contribute to the floral character of the grapes [4]. Consistent QTLs with minor contributions were also detected in both years for phenethyl alcohol and cis-3-nonen-1-ol. Then, significant differences in the concentrations of these volatiles among the genotypes of the QTLs were confirmed (Table 1; Figs. 5 and 6h, i). Additionally, several other minor QTLs were detected in lactones, alcohols, and shikimic acid derivatives during either of the 2 years (Table S5). These findings suggest these volatiles were regulated by multiple loci acting in combination, which alone only has minor influence. The involvement of several minor QTLs for similar groups of volatiles, such as lactones, alcohols, esters and benzenes, has also been reported in other fruits, supporting the multigenic inheritance, particularly of the volatiles in these groups [36–38].

Wu et al. [11] previously classified the aroma types of table grapes, including the American hybrids, into three types: “strawberry”, “foxy” and “muscat”. While MA belongs to the “muscat” aroma type, CE is considered to belong to the “strawberry” or “foxy” aroma type. Furthermore, among the hybrid population, the profile of methyl anthranilate and *o*-aminoacetophenone in C5, which contributes to the “foxy” aroma, was distinct from that of mesifurane in C7, influencing the “strawberry” aroma (Fig. 4). The average concentration of free mesifurane among Pop AC was 10.6 μg/kg berries. Since mesifurane showed very low sensory thresholds of 0.03 μg/kg berries [39], the concentration of Pop AC was above the threshold, therefore, contributing to the strawberry aroma of the berries. However, although furaneol was reportedly contained at higher concentrations than mesifurane in mature berries [9], furaneol was undetected in the hybrid population in this study. The reason why furaneol was undetected was unclear. However, it is possible that the high hydrophilic nature of this compound made the extraction by the SBSE twister difficult in our analytical system. Additionally, consistent with its distinct profile among Pop AC, we could detect a specific QTL for mesifurane on LG12 in the CE maps, different from that found for *o*-aminoacetophenone on LG3 (Additional File 3: Fig. S3, Additional file 4: Table S5). Thus, these results suggest the different genetic regulation of the aroma types of table grapes.

Although the wide variations of berry aroma compound profile in our population enabled us to understand the overviews of the genetic architecture in interspecific hybrid grapes, further researches aiming to improve the accuracy of mapped QTLs by increasing the number of individuals, and by using different biparental populations are necessary to identify the stable QTLs and candidate gene, especially for the QTLs that were newly found in this study.

## Conclusions

From the cluster analysis of volatile compounds using a grape QTL mapping population between *V. labruscana* ‘Campbell Early’ and *V. vinifera* ‘Muscat of Alexandria’, we showed a group of volatile compounds that share biosynthetic pathways were classified into the same or close clusters. This result suggests a common mechanism for controlling their concentrations in each group. Furthermore, specific QTLs obtained for some of these groups supported the hypothesis that the accumulation of each group of volatiles was genetically controlled independently. For example, in addition to the reported major QTL on LG5, which controls the concentrations of monoterpenes, several QTLs (on LG2, LG13 and LG15) were identified to affect each monoterpene specifically, modulating their composition. A major QTL on LG2 was also found newly for norisoprenoid concentrations, showing distinct genetic regulation from monoterpenes. Moreover, the genetic loci related to the characteristics of American hybrid volatiles, including *o*-aminoacetophenone and mesifurane, were different from those of monoterpenes and norisoprenoids. Therefore, the discovered genetic architecture of volatilomes in grapes suggested the different genetic regulation for the aroma types in the interspecific hybrid grapes.

## Methods

### Plant materials

The mapping population used in this study (Pop AC, 95 F_1_ individuals) was generated by crossing MA and CE from 2007 to 2010. Individuals of the population were grafted onto Kober 5BB rootstocks and planted in a vineyard at the Grape and Persimmon Research Station, NARO, Higashihiroshima, Japan. Phenotypic data used were obtained over 2 years (2014 and 2015). We observed that 82 and 90 individuals bore fruit in 2014 and 2015, respectively. Subsequently, fruit clusters from each individual were harvested when they were fully ripen, based on sensory evaluation, as described previously [40]. Clusters from the parent cultivars were also harvested for comparison. Fifteen berries from each individual were randomly collected from three clusters, after which they were deseeded. After deseeding, the samples were immediately frozen in liquid nitrogen and stored at − 80 °C until further analysis. Simultaneously, meteorological data of the region, such as the average temperature, total rainfall and total sunshine duration per month during the developmental period, were taken for 2 years from the Automated Meteorological Data Acquisition System database of the Japan Meteorological Agency (Table S2).

### Chemicals and reagents

The volatile standards used in this study are shown in Additional File 4: Table S1. First, 2,4-dichloroaniline (99.5% purity) was purchased from Tokyo Chemical Industry Co., Ltd. (Tokyo, Japan) for the reagents. However, while n-heptyl β-D-glucopyranoside (98% purity) was purchased from Sigma-Aldrich, Inc. (St. Louis, MO, USA), dichloromethane (99.5% purity), liquid chromatography-mass spectrometry-grade methanol, citric acid, sodium phosphates, sodium fluoride (NaF), ascorbic acid, sodium hydroxide (NaOH) and 3-octanol (97% purity) were obtained from Wako Co. Ltd. (Osaka, Japan).

### Analysis of bound and free volatile compounds

According to a previously described procedure, extraction, fractionation and quantification of bound and free volatiles in the berries were performed [29]. Briefly, 15 whole berries from each sample were partially crushed in liquid nitrogen using a mortar and pestle. Then, three replicate portions from a pool were powdered in a bead mill (Yasui Kikai, Osaka, Japan), after which volatile fractions were extracted in 0.13 M NaF and 50 mg/L ascorbic acid solution by shaking for 15 min at 4 °C. Next, extracts with n-heptyl β-D-glucopyranoside as the surrogate standard were added to the solid-phase extraction column, LiChrolut-EN cartridge (Merck, Darmstadt, Germany), after which the resulting free and bound volatile fractions were collected. Subsequently, the eluate of the bound volatile fraction was divided into two parts: enzymatic and heat-acid hydrolysis. Afterward, while the AR 2000 pectinase enzyme (DMS Food Specialties Beverages Ingredients, Delft, The Netherlands) catalysed enzymatic hydrolysis with β-glucosidase activity, heat-acid hydrolysis was performed in a water bath heated to 100 °C for 1 h to analyze the bound norisoprenoids in an encapsulated vial at pH 2.5 under a nitrogen atmosphere. Then, 10 mL of the extracted eluent containing released volatile compounds was dispensed into a 10-mL glass headspace vial with 2 g NaCl and 0.5 mg/L 3-octanol, used as the internal standard. A stir bar (Twister from Gerstel, Mulhein an der Ruhr, Germany, 10-mm length, 0.5-mm layer) was later placed in, and the mixture was stirred at 22 °C for 4 h. After, the stir bar was removed and transferred to a glass thermal desorption tube for GCMS analysis. For free volatile analyses, the dichloromethane in the eluent was evaporated to replace with the water before the SBSE extraction using the procedure above. Finally, as described previously, chromatographic analysis was performed by GCMS with three replicate samples for volatile compound identification/quantification [29].

### Analysis of free volatiles with trace levels

Free volatiles with trace levels, which SBSE-GCMS could not analyse, were analysed by using the SPME-GCMSMS method. First, powdered samples ground from the whole berries without seeds were mixed with half the weight of 0.1 M EDTA/NaOH (pH 7.5), after which CaCl_2_ was added to a final concentration of 5% (w/w). Then, the mixture was shaken for 30 min at 4 °C. Next, 8.5 g of the homogenate was dispensed into a 20-mL amber SPME vial, with 3 g of NaCl and 5 μg/kg 2,4-dichloroaniline as the internal standards. SPME vials were incubated and equilibrated at 80 °C for 10 min under continuous agitation at 450 rpm. Subsequently, volatile compounds were extracted by inserting a two-phase SPME fiber and 50/30 μm divinylbenzene-polydimethylsiloxane (Supelco, Bellafonte, PA, USA) into the headspace of the vial for 60 min under the same condition. After, GCMSMS analyses were performed using a gas chromatograph of a Shimadzu GC-2010 Plus coupled with a TQ8040 mass spectrometer (Shimadzu Corporation, Kyoto, Japan). Analyte separation was achieved using a DB-5 column (30 m × 0.25 mm × 1 μm film thickness; Agilent Technologies Inc., Wilmington, DE, USA). First, the SPME fiber was thermally desorbed in a GC injection port at 250 °C for 10 min (splitless injection). High-purity helium was used as a carrier at a 1.0 mL/min constant flow rate. Then, the GC oven temperature was programmed to start at 40 °C, with a 5-min hold, then increase to 94 °C at 4 °C/min, 160 °C at 2 °C/min, 260 °C at 50 °C/min and to hold again at 260 °C for 5 min. The MS was operated in an electron ionisation mode at 70 eV. The emission current was 150 μA, and the ion source and transfer line temperatures were 200 °C and 250 °C, respectively. High-purity argon was used as the collision gas. Furthermore, mass spectra were acquired in multiple reaction monitoring (MRM) modes to quantify trace volatiles and 2,4-dichloroaniline, the internal standard, with a dwell time of 0.050 min. The detailed MRM conditions and collision energy (CE) for each compound were as follows: For methyl anthranilate, the transitions were m/z 151 to 119 and m/z 119 to 92, both with CE at 10 V; for *o*-aminoacetophenone, the transitions were m/z 135 to 120 and m/z 120 to 92, both with CE at 10 V. However, for 1,8-cineol, the transitions were m/z 154 to 69 (15 V) and m/z 154 to 125 (5 V), and for 2,4-dichloroaniline, the transitions were m/z 161 to 90 (15 V) and m/z 161 to 125 (10 V). The first transition was used for the quantification, while the second was used as a qualifier.

### QTL analysis of the volatiles

QTL analysis was conducted using both parental and consensus maps in the MapQTL v. 6 software [41]. The details of the genetic linkage maps of Pop AC have been described previously [27]. Then, newly developed SSRs on LG5 (two SSRs), LG10 (10 SSRs) and LG11 (three SSRs) were added to the map in this study. Primers for these SSRs are shown in Additional file 4: Table S6. Subsequently, the volatile data from individuals (82 in 2014; 90 in 2015) for each year and the mean values over the 2 years were subjected to QTL analysis. QTLs were identified by interval mapping, after which the LOD threshold corresponding to a genome-wide significance level of 0.05 was determined using 1000 permutation cycles. Later, QTLs were selected on the basis of the LOD peak scores exceeding a threshold value, after which the confidence interval based on one LOD was used to estimate the putative QTL position. Then, a consistent QTL detected reproducibly over 2 years were selected and confirmed by the data averaged over 2 years. The QTL locations on the linkage maps were illustrated using MapChart 2.32 software [42]. Finally, to confirm the QTL’s effect, the relationship between the phenotypic values for 2 years and the genotypes of the markers close to the QTL were analysed.

Subsequently, a 12 × PN40024 genomic sequence [17] was used to identify potential candidate genes underlying QTLs near SSR markers. Then, the physical locations of candidate genes on their biosynthetic aroma compound pathway were finally compared with those of the SSR markers underlying QTLs by the NCBI Map Viewer public database.

### Statistical analyses

Student’s *t*-test was used to evaluate the differences between each volatile concentration in the berries of the parent cultivars. Hierarchical clustering was conducted for volatile data from Pop AC to group them by volatiles and genotypes. A one-way analysis of variance and Tukey’s honest significant difference (HSD) were performed to compare the concentrations of the volatiles for the genotypes of SSR markers nearest to the identified QTLs. These statistical analyses, including histogram, box plot, and heat map visualisation, were performed using the JMP software, version 14.0 (SAS Institute Inc., Cary, NC, USA). PCA was also performed to compare the volatile profiles of genotypes and vintages. This analysis was performed using the SIMCA software, version 15 (MKS Umetrics, Malmo, Sweden).

## Supplementary Information

**Additional file 1: Fig. S1**. Volatile compositions in ‘Muscat of Alexandria’ and ‘Campbell Early’ berries. Both the bound and free volatiles detected were classified into nine chemical groups. Then, the total concentrations of each group averaged over 2 years (a), and the concentrations of individual bound (b) and free (c) monoterpenes of the two cultivars, were shown as pie charts for the comparison.

**Additional file 2: Fig. S2**. Histograms of volatile concentrations of the hybrid population (Pop AC) for the 2 years. Histograms of 45 volatiles for the 2 years (2014 and 2015) are shown ((a)-(as)). The concentrations of the volatiles are expressed as μg per g of berries. The frequency of a given class for the population is shown on the vertical axis. Arrows indicate the concentration positions of the two-parent cultivars; MA: ‘Muscat of Alexandria’, CE: ‘Campbell Early’. The coefficient of determination between years for each volatile is shown in each histogram’s upper right section.

**Additional file 3: Fig. S3**. Box plot showing the volatiles concentrations for the genotypes of SSR markers and MYB haplotypes nearest to the QTL in the hybrid population (Pop AC). Genotypes of the SSR markers were expressed as allele lengths in bp. Horizontal lines inside boxes show median values, whereas horizontal lines through boxes indicate means. Box height indicate 50% of the data. Different letters (α, β, γ) indicate that the values are significantly different at *P* < 0.05 by Tukey’s HSD. MA: ‘Muscat of Alexandria’, CE: ‘Campbell Early’.

**Additional file 4: Table S1**. Free and bound volatile components identified in ‘Muscat of Alexandria’ and ‘Campbell Early’ berries. **Table S2**. Meteorological conditions in the Higashihiroshima region during the developmental period of grape berries in 2014 and 2015. **Table S5**. QTL analysis results showing the hybrid population’s volatile concentrations (Pop AC). **Table S6**. Newly designed primers used in this study.

**Additional file 5: Table S3**. The correlation matrix showing the concentrations of 45 volatiles in 2014 among the hybrid population (Pop AC).

**Additional file 6: Table S4**. The correlation matrix showing the concentrations of 45 volatiles  in 2015 among the hybrid population (Pop AC).

## Data Availability

All the relevant data and supporting materials can be found in the paper.

## Abbreviations

Bp: Base pair
CA: ‘Campbell Early’
CE: Collision energy
GCMS: Gas chromatography-mass spectrometry
MEP pathway: Methyl-erythritol-phosphate pathway
MA: ‘Muscat of Alexandria’
MRM: Multiple reaction monitoring
MSMS: Tandem mass spectrometry
LG: Linkage group
PC: Principal component
PCA: Principal component analysis
SBSE: Stir bar sorptive extraction
SPME: Solid-phase microextraction
Tukey’s HSD: Tukey’s honest significant difference
QTL: Quantitative trait locus
SSR: Simple sequence repeats
