# Supplementary material for: Genetic architecture of berry aroma compounds in a QTL (quantitative trait loci) mapping population of interspecific hybrid grapes (Vitis labruscana × Vitis vinifera)
Source: BMC Plant Biol. 2022 Sep 23;22:458. doi: 10.1186/s12870-022-03842-z (PMC9503205; doi:10.1186/s12870-022-03842-z)
Supplement: Supplementary file 1 — Additional file 1: Fig. S1. Volatile compositions in ‘Muscat of Alexandria’ and ‘Campbell Early’ berries. Both the bound and free volatiles detected were classified into nine chemical groups. Then, the total concentrations of each group averaged over 2 years (a), and the concentrations of individual bound (b) and free (c) monoterpenes of the two cultivars, were shown as pie charts for the comparison. [file 12870_2022_3842_MOESM1_ESM.pptx]

## Slide 1
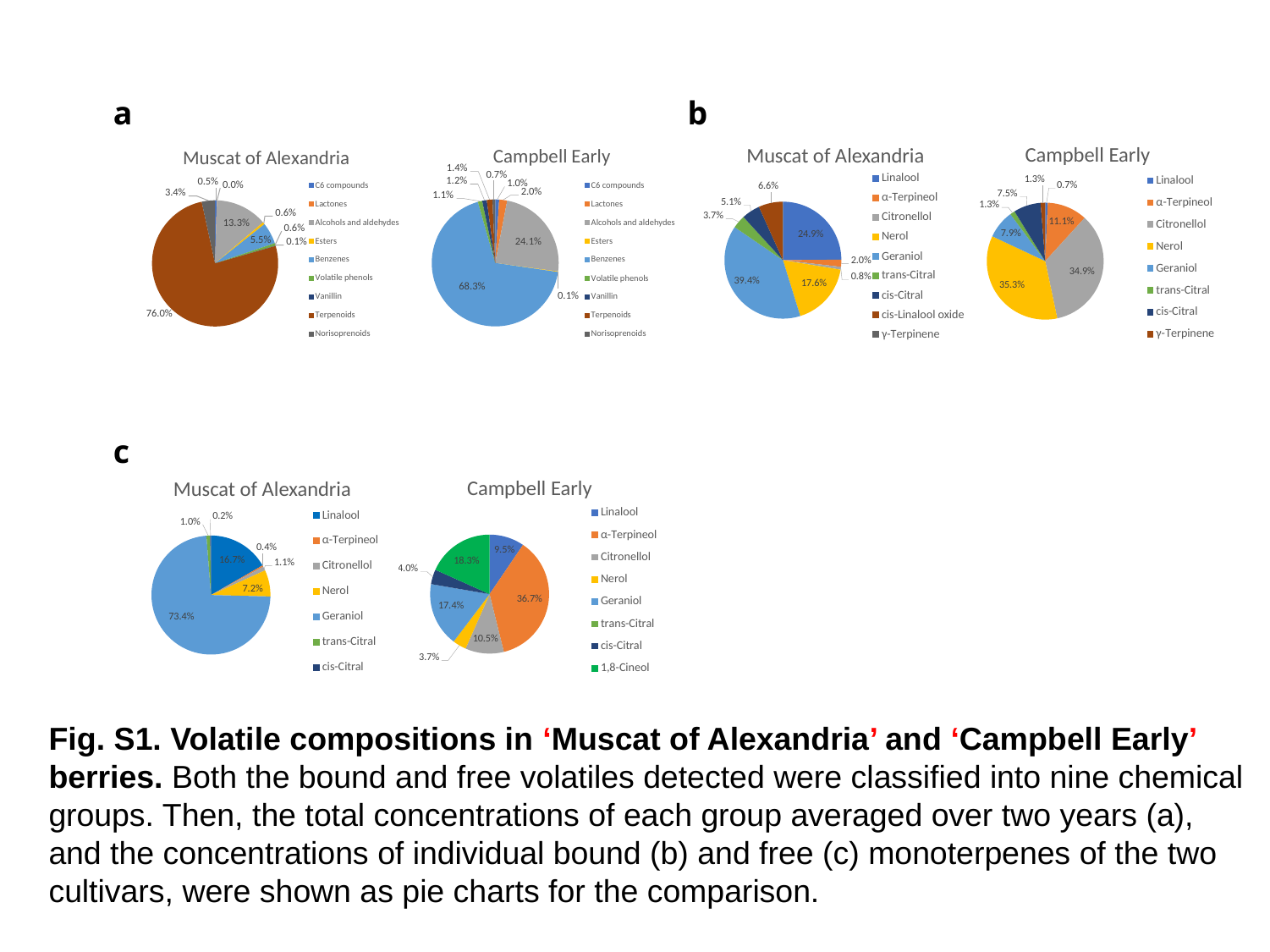

a
b
c
Fig. S1. Volatile compositions in ‘Muscat of Alexandria’ and ‘Campbell Early’ berries. Both the bound and free volatiles detected were classified into nine chemical groups. Then, the total concentrations of each group averaged over two years (a), and the concentrations of individual bound (b) and free (c) monoterpenes of the two cultivars, were shown as pie charts for the comparison.
