# Supplementary material for: Genetic architecture of berry aroma compounds in a QTL (quantitative trait loci) mapping population of interspecific hybrid grapes (Vitis labruscana × Vitis vinifera)
Source: BMC Plant Biol. 2022 Sep 23;22:458. doi: 10.1186/s12870-022-03842-z (PMC9503205; doi:10.1186/s12870-022-03842-z)
Supplement: Supplementary file 2 — Additional file 2: Fig. S2. Histograms of volatile concentrations of the hybrid population (Pop AC) for the 2 years. Histograms of 45 volatiles for the 2 years (2014 and 2015) are shown ((a)-(as)). The concentrations of the volatiles are expressed as μg per g of berries. The frequency of a given class for the population is shown on the vertical axis. Arrows indicate the concentration positions of the two-parent cultivars; MA: ‘Muscat of Alexandria’, CE: ‘Campbell Early’. The coefficient of determination between years for each volatile is shown in each histogram’s upper right section. [file 12870_2022_3842_MOESM2_ESM.pptx]

## Slide 1
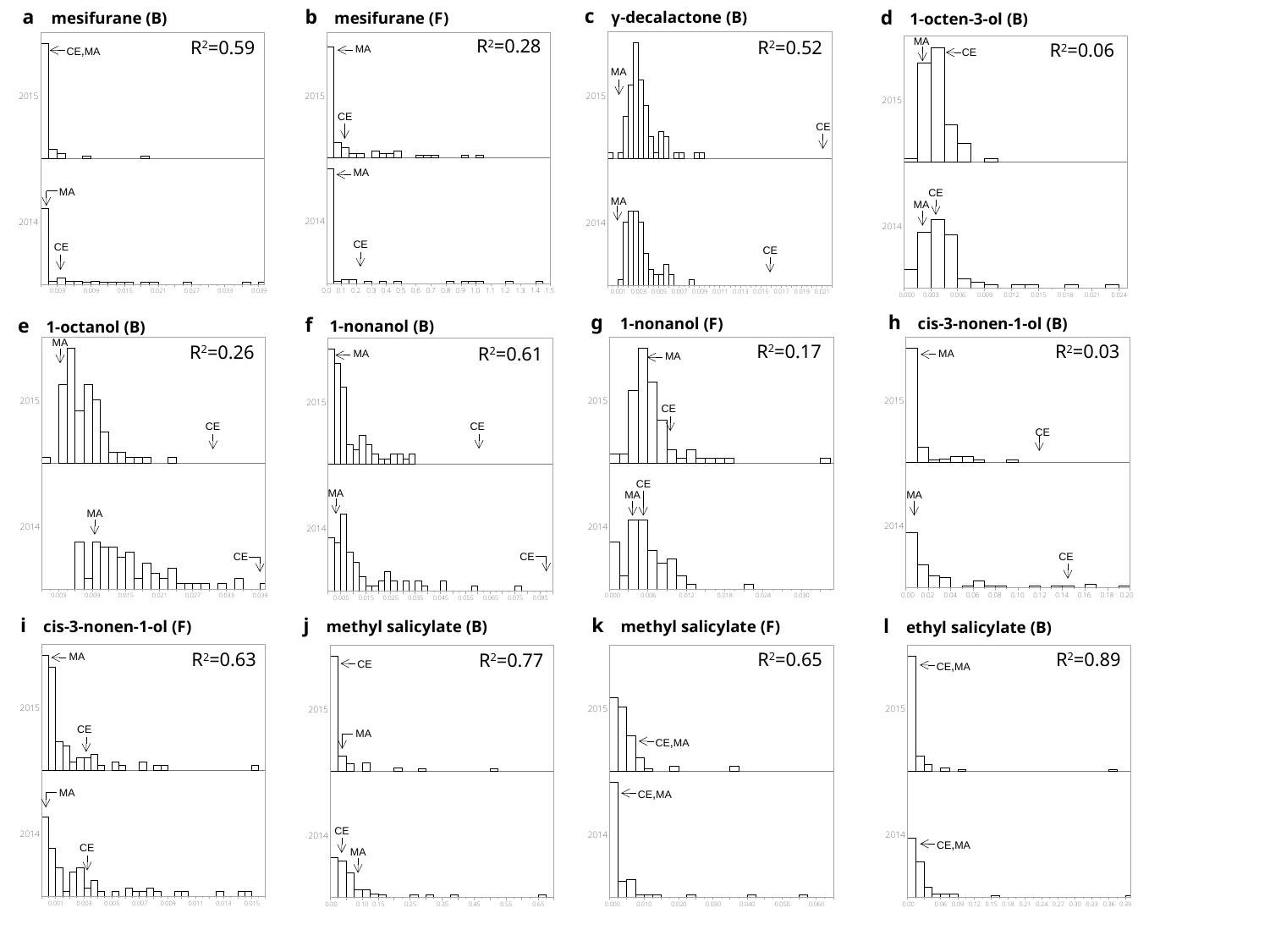

c γ-decalactone (B)
a mesifurane (B)
b mesifurane (F)
d 1-octen-3-ol (B)
MA
R2=0.28
R2=0.59
R2=0.52
R2=0.06
MA
CE,MA
CE
MA
CE
MA
CE
CE
MA
MA
CE
MA
MA
CE
CE
CE
g 1-nonanol (F)
h cis-3-nonen-1-ol (B)
f 1-nonanol (B)
e 1-octanol (B)
MA
R2=0.17
R2=0.03
R2=0.26
R2=0.61
MA
MA
MA
CE
CE
CE
CE
CE
MA
MA
MA
MA
CE
CE
CE
i cis-3-nonen-1-ol (F)
j methyl salicylate (B)
k methyl salicylate (F)
l ethyl salicylate (B)
R2=0.65
R2=0.89
R2=0.63
R2=0.77
MA
CE
CE,MA
CE
MA
CE,MA
MA
CE,MA
CE
CE,MA
CE
MA

## Slide 2
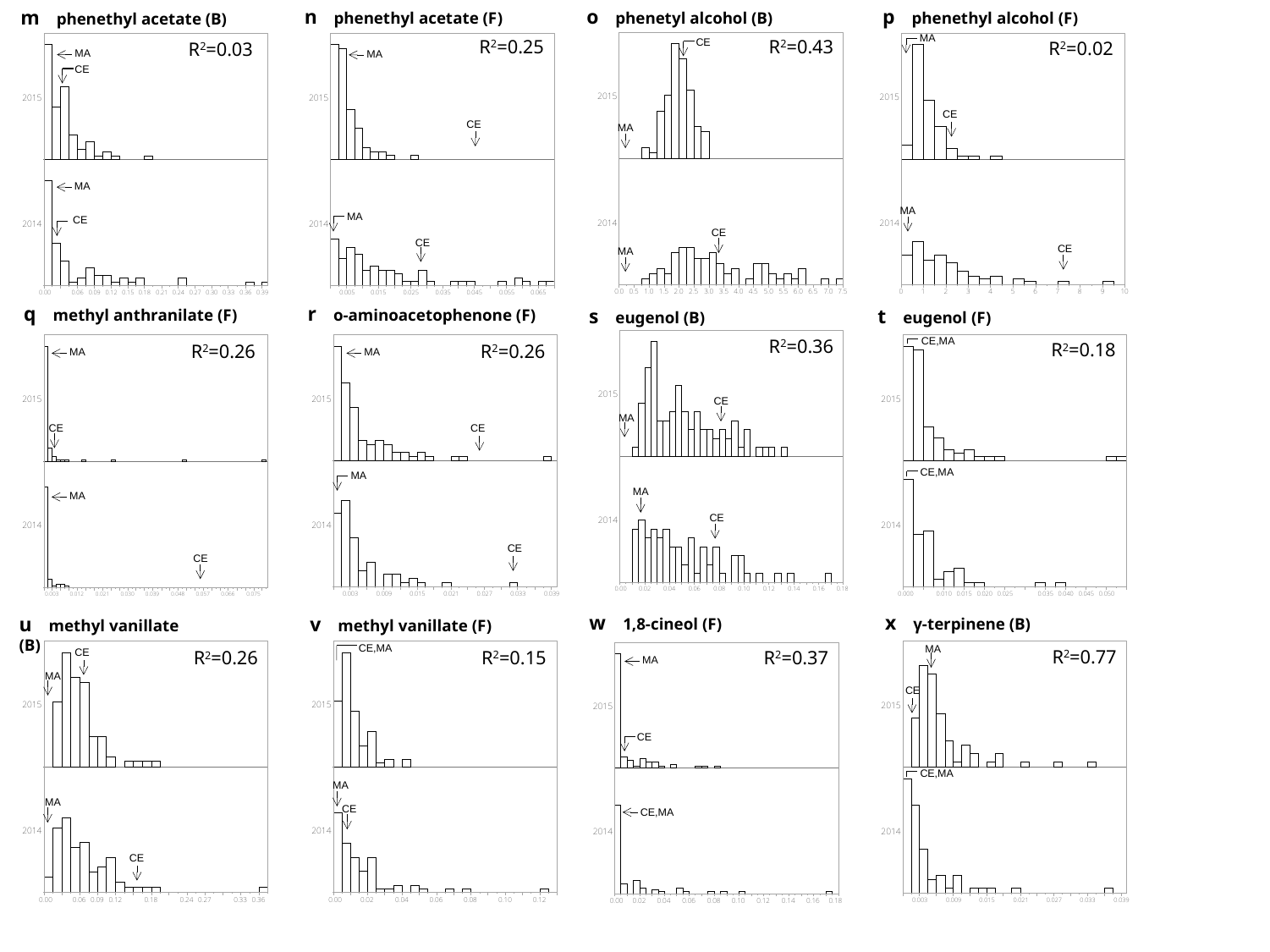

n phenethyl acetate (F)
o phenetyl alcohol (B)
p phenethyl alcohol (F)
m phenethyl acetate (B)
MA
R2=0.25
R2=0.43
CE
R2=0.02
R2=0.03
MA
MA
CE
CE
CE
MA
MA
MA
MA
CE
CE
CE
CE
MA
q methyl anthranilate (F)
r o-aminoacetophenone (F)
s eugenol (B)
t eugenol (F)
CE,MA
R2=0.36
R2=0.18
R2=0.26
R2=0.26
MA
MA
CE
MA
CE
CE
CE,MA
MA
MA
MA
CE
CE
CE
w 1,8-cineol (F)
x γ-terpinene (B)
u methyl vanillate (B)
v methyl vanillate (F)
CE,MA
MA
R2=0.77
CE
R2=0.15
R2=0.37
R2=0.26
MA
MA
CE
CE
CE,MA
MA
MA
CE
CE,MA
CE

## Slide 3
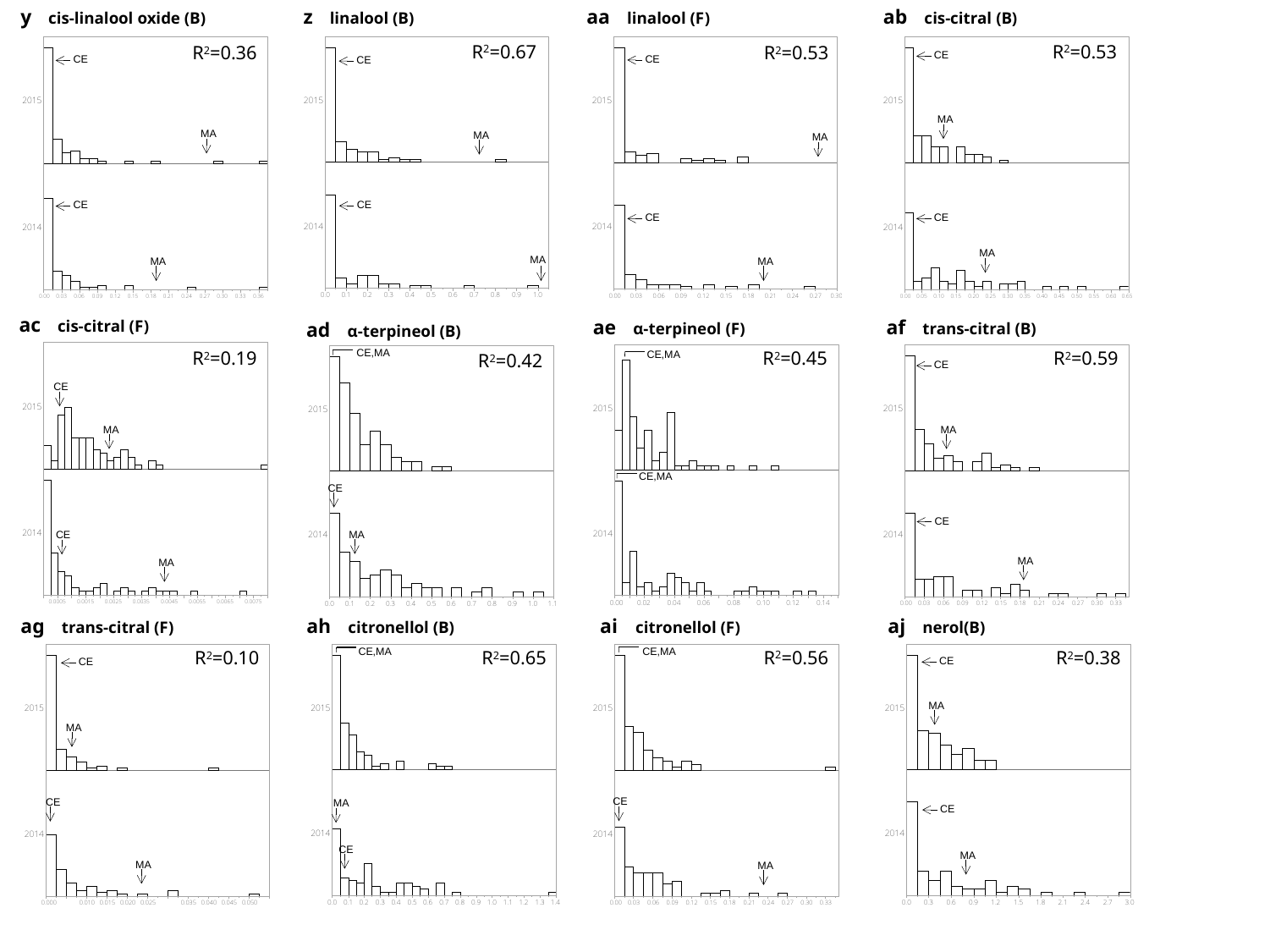

aa linalool (F)
ab cis-citral (B)
y cis-linalool oxide (B)
z linalool (B)
R2=0.53
R2=0.67
R2=0.53
R2=0.36
CE
CE
CE
CE
MA
MA
MA
MA
CE
CE
CE
CE
MA
MA
MA
MA
ac cis-citral (F)
ae α-terpineol (F)
af trans-citral (B)
ad α-terpineol (B)
CE,MA
R2=0.59
R2=0.45
R2=0.19
CE,MA
R2=0.42
CE
CE
MA
MA
CE,MA
CE
CE
MA
CE
MA
MA
ag trans-citral (F)
ah citronellol (B)
ai citronellol (F)
aj nerol(B)
CE,MA
CE,MA
R2=0.56
R2=0.65
R2=0.10
R2=0.38
CE
CE
MA
MA
CE
CE
MA
CE
CE
MA
MA
MA

## Slide 4
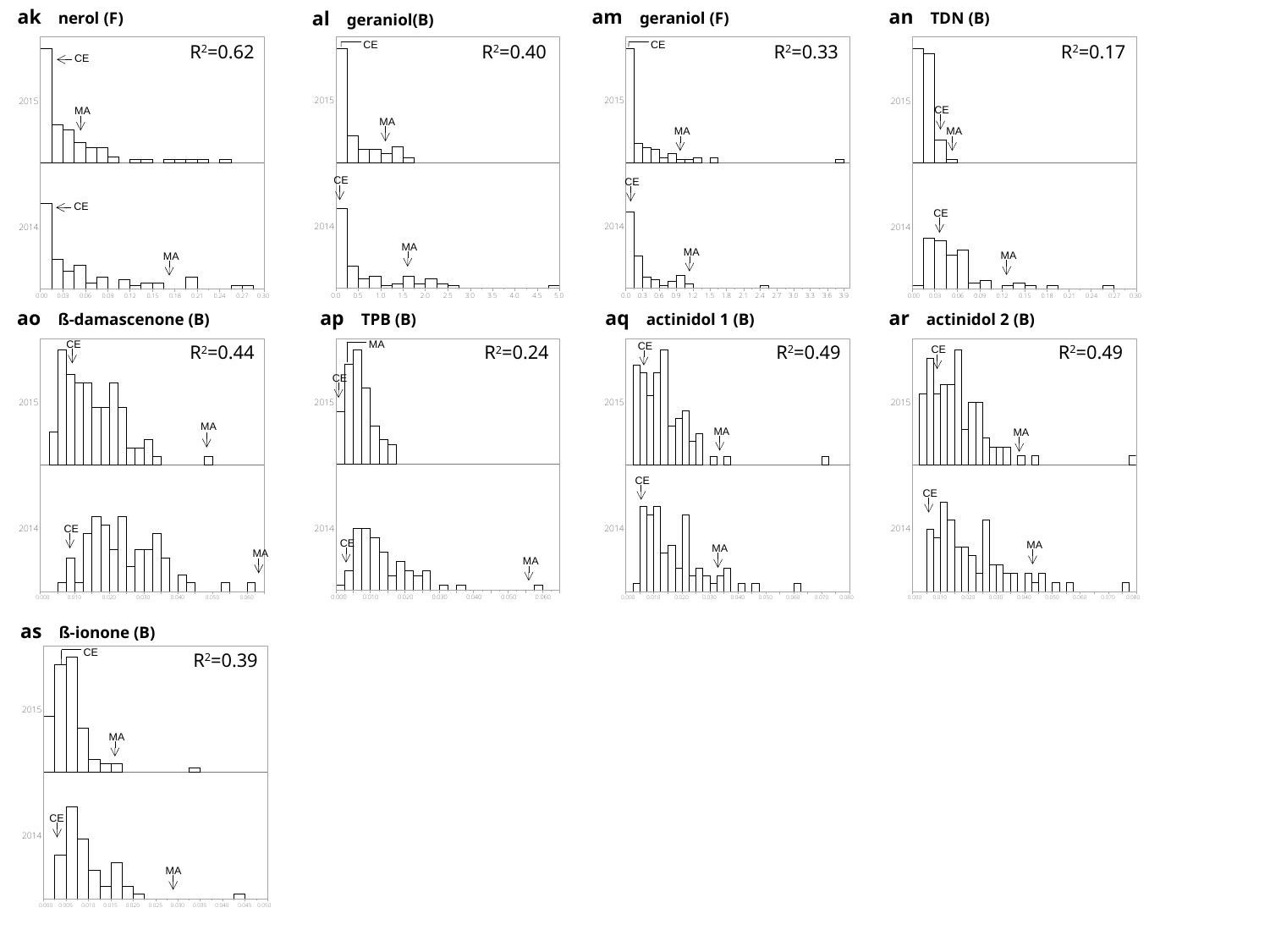

ak nerol (F)
am geraniol (F)
an TDN (B)
al geraniol(B)
CE
CE
R2=0.40
R2=0.62
R2=0.17
R2=0.33
CE
CE
MA
MA
MA
MA
CE
CE
CE
CE
MA
MA
MA
MA
ao ß-damascenone (B)
ap TPB (B)
aq actinidol 1 (B)
ar actinidol 2 (B)
CE
MA
CE
R2=0.49
R2=0.49
R2=0.44
R2=0.24
CE
CE
MA
MA
MA
CE
CE
CE
CE
MA
MA
MA
MA
as ß-ionone (B)
CE
R2=0.39
MA
CE
MA

## Slide 5
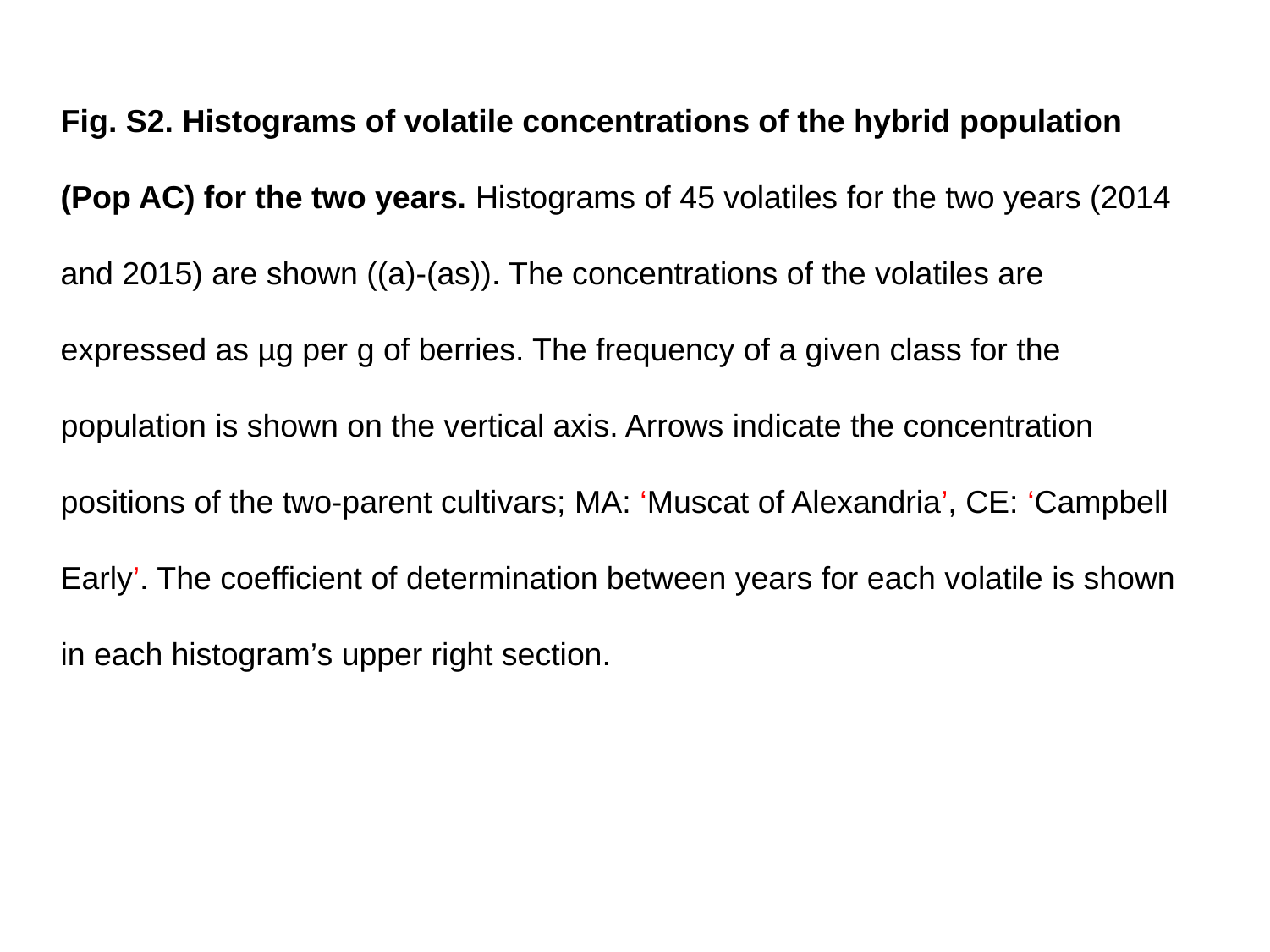

Fig. S2. Histograms of volatile concentrations of the hybrid population (Pop AC) for the two years. Histograms of 45 volatiles for the two years (2014 and 2015) are shown ((a)-(as)). The concentrations of the volatiles are expressed as µg per g of berries. The frequency of a given class for the population is shown on the vertical axis. Arrows indicate the concentration positions of the two-parent cultivars; MA: ‘Muscat of Alexandria’, CE: ‘Campbell Early’. The coefficient of determination between years for each volatile is shown in each histogram’s upper right section.
