# Supplementary material for: Genetic architecture of berry aroma compounds in a QTL (quantitative trait loci) mapping population of interspecific hybrid grapes (Vitis labruscana × Vitis vinifera)
Source: BMC Plant Biol. 2022 Sep 23;22:458. doi: 10.1186/s12870-022-03842-z (PMC9503205; doi:10.1186/s12870-022-03842-z)
Supplement: Supplementary file 3 — Additional file 3: Fig. S3. Box plot showing the volatiles concentrations for the genotypes of SSR markers and MYB haplotypes nearest to the QTL in the hybrid population (Pop AC). Genotypes of the SSR markers were expressed as allele lengths in bp. Horizontal lines inside boxes show median values, whereas horizontal lines through boxes indicate means. Box height indicate 50% of the data. Different letters (α, β, γ) indicate that the values are significantly different at P < 0.05 by Tukey’s HSD. MA: ‘Muscat of Alexandria’, CE: ‘Campbell Early’. [file 12870_2022_3842_MOESM3_ESM.pptx]

## Slide 1
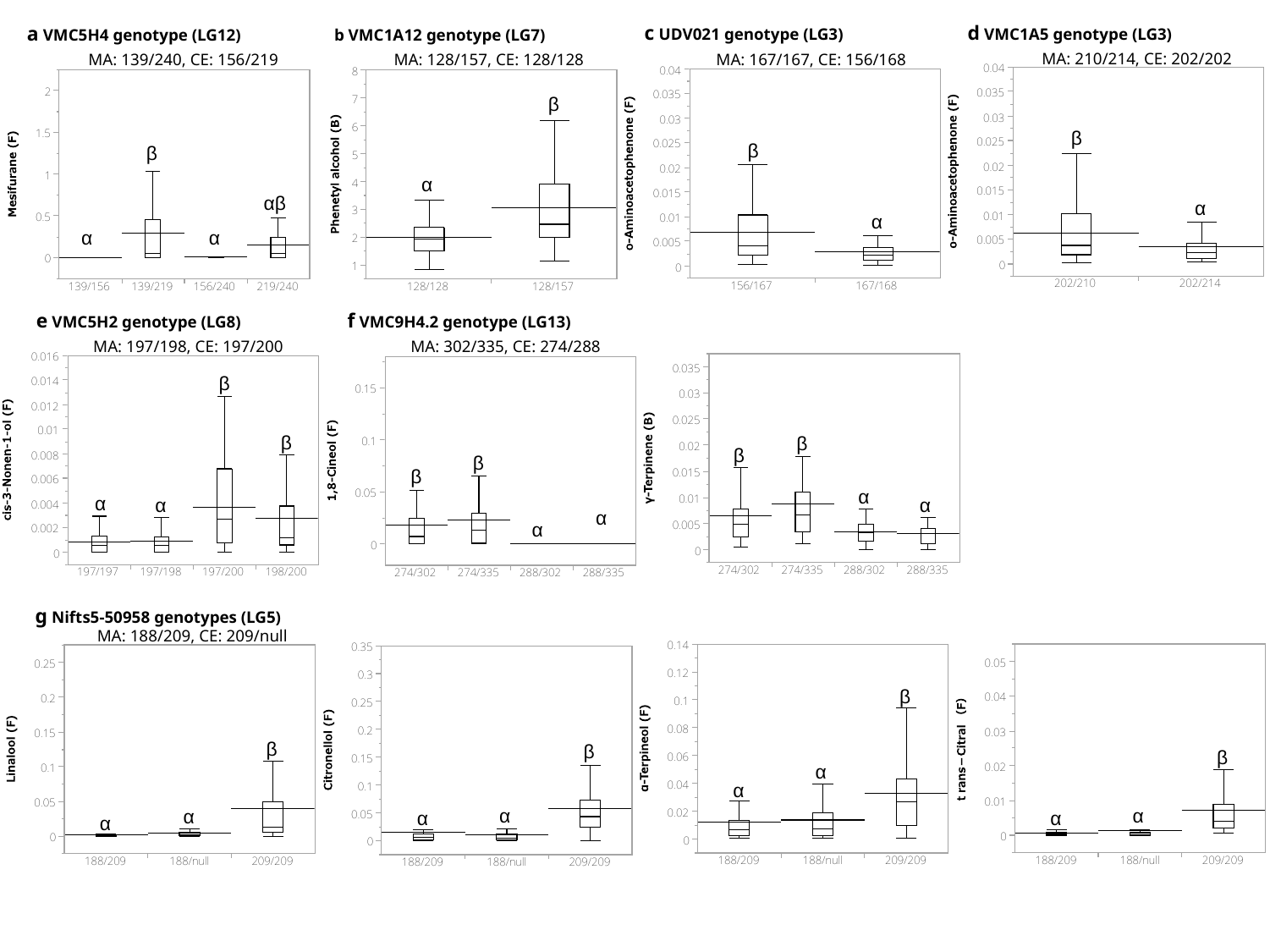

d VMC1A5 genotype (LG3)
c UDV021 genotype (LG3)
a VMC5H4 genotype (LG12)
b VMC1A12 genotype (LG7)
MA: 210/214, CE: 202/202
MA: 128/157, CE: 128/128
MA: 167/167, CE: 156/168
MA: 139/240, CE: 156/219
β
β
β
β
α
αβ
α
α
α
α
e VMC5H2 genotype (LG8)
f VMC9H4.2 genotype (LG13)
MA: 197/198, CE: 197/200
MA: 302/335, CE: 274/288
β
β
β
β
β
β
α
α
α
α
α
α
g Nifts5-50958 genotypes (LG5)
MA: 188/209, CE: 209/null
β
β
β
β
α
α
α
α
α
α
α
α

## Slide 2
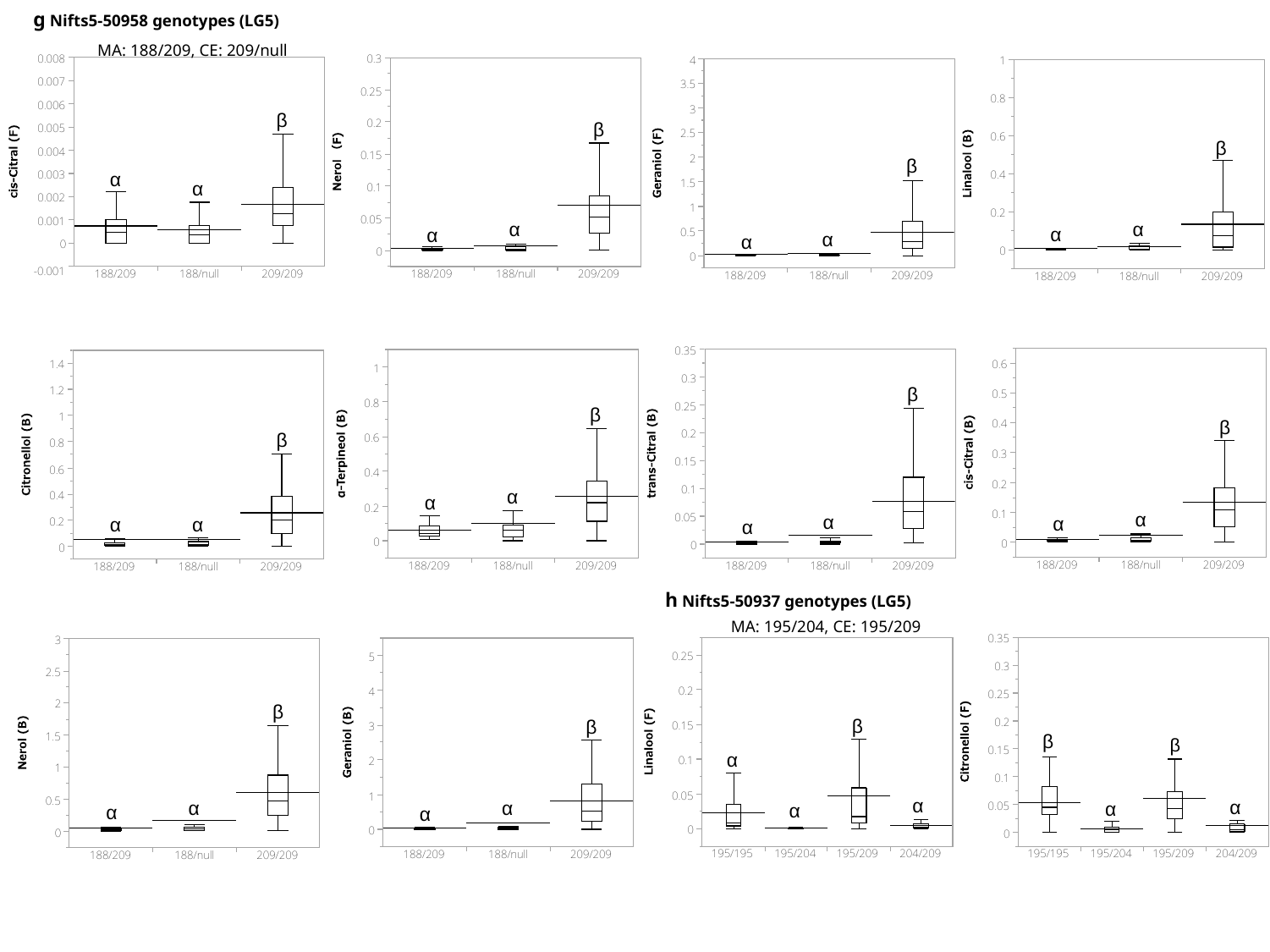

g Nifts5-50958 genotypes (LG5)
MA: 188/209, CE: 209/null
β
β
β
β
α
α
α
α
α
α
α
α
β
β
β
β
α
α
α
α
α
α
α
α
h Nifts5-50937 genotypes (LG5)
MA: 195/204, CE: 195/209
β
β
β
β
β
α
α
α
α
α
α
α
α
α

## Slide 3
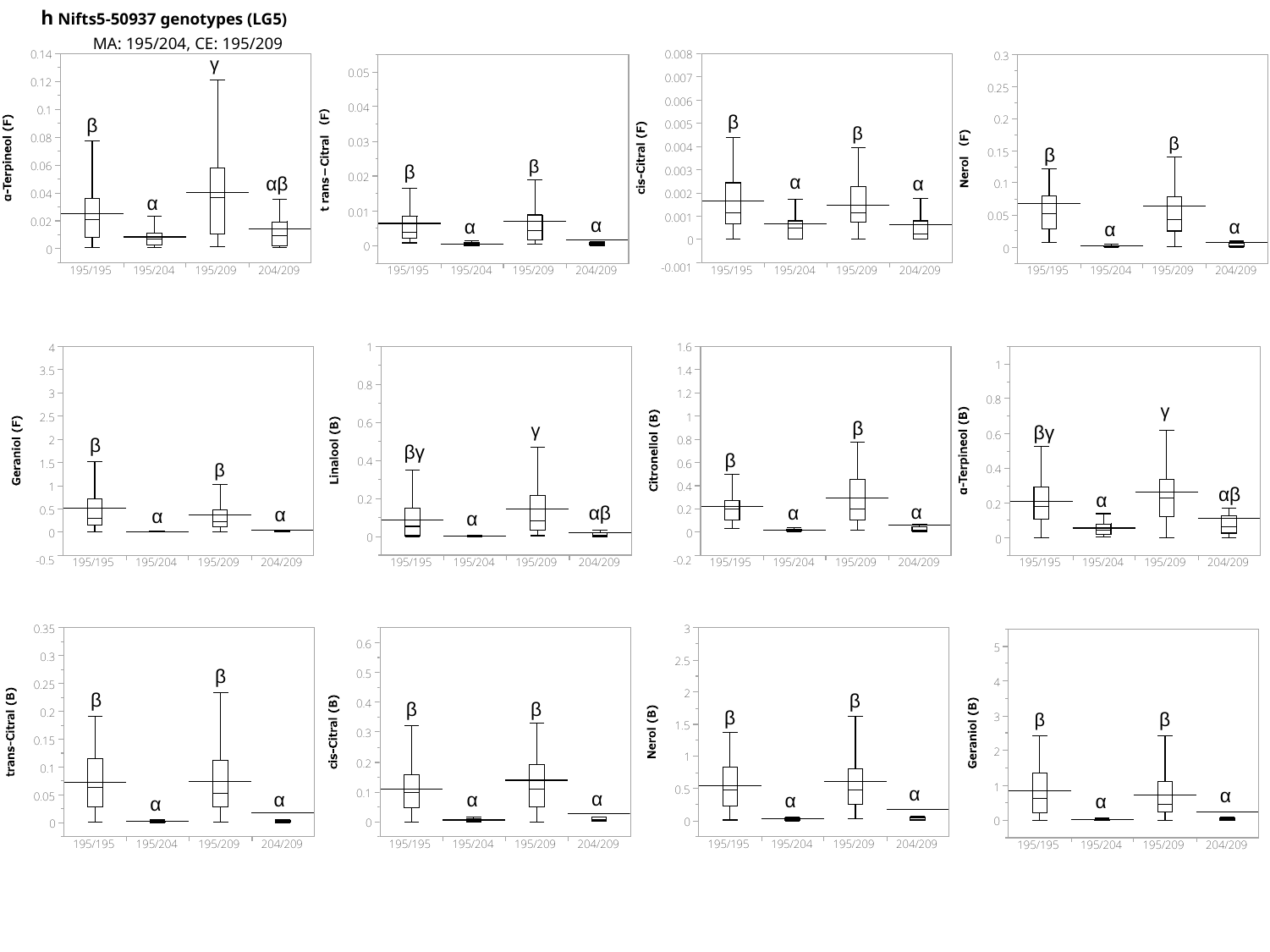

h Nifts5-50937 genotypes (LG5)
MA: 195/204, CE: 195/209
γ
β
β
β
β
β
β
β
α
αβ
α
α
α
α
α
α
γ
β
γ
βγ
β
βγ
β
β
αβ
α
α
α
αβ
α
α
α
β
β
β
β
β
β
β
β
α
α
α
α
α
α
α
α

## Slide 4
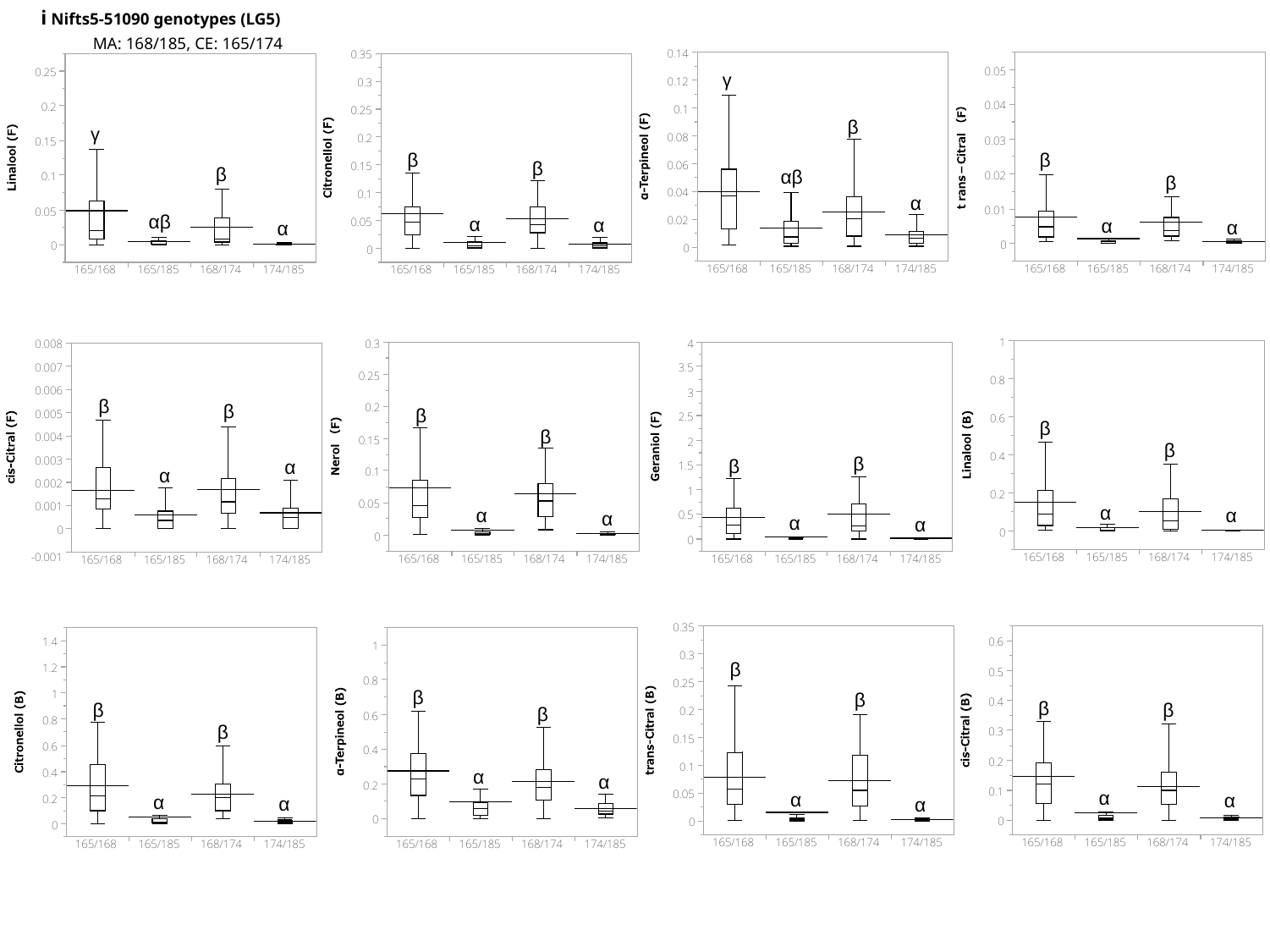

i Nifts5-51090 genotypes (LG5)
MA: 168/185, CE: 165/174
γ
β
γ
β
β
β
β
αβ
β
α
αβ
α
α
α
α
α
β
β
β
β
β
β
β
β
α
α
α
α
α
α
α
α
β
β
β
β
β
β
β
β
α
α
α
α
α
α
α
α

## Slide 5
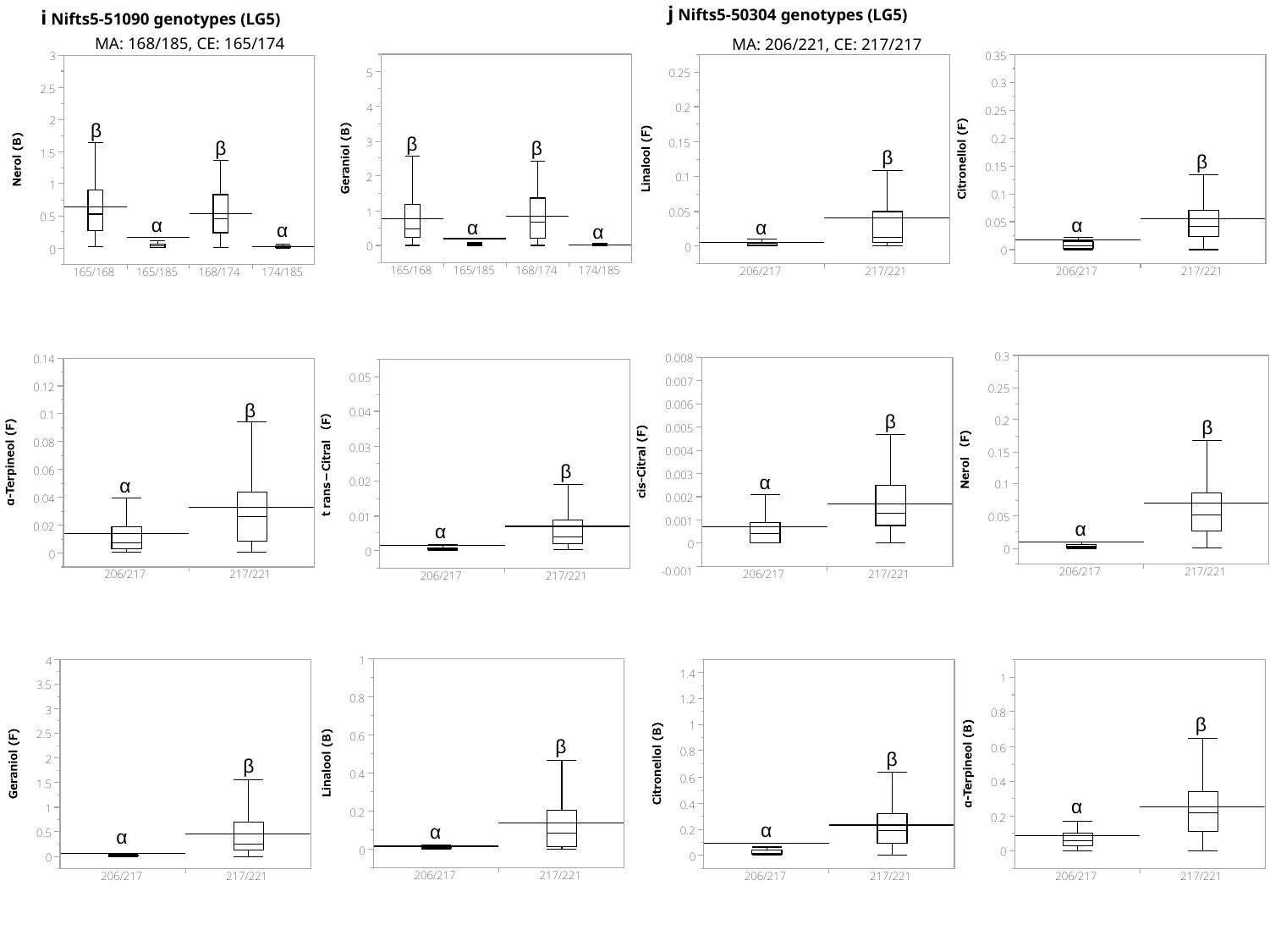

j Nifts5-50304 genotypes (LG5)
i Nifts5-51090 genotypes (LG5)
MA: 168/185, CE: 165/174
MA: 206/221, CE: 217/217
β
β
β
β
β
β
α
α
α
α
α
α
β
β
β
β
α
α
α
α
β
β
β
β
α
α
α
α

## Slide 6
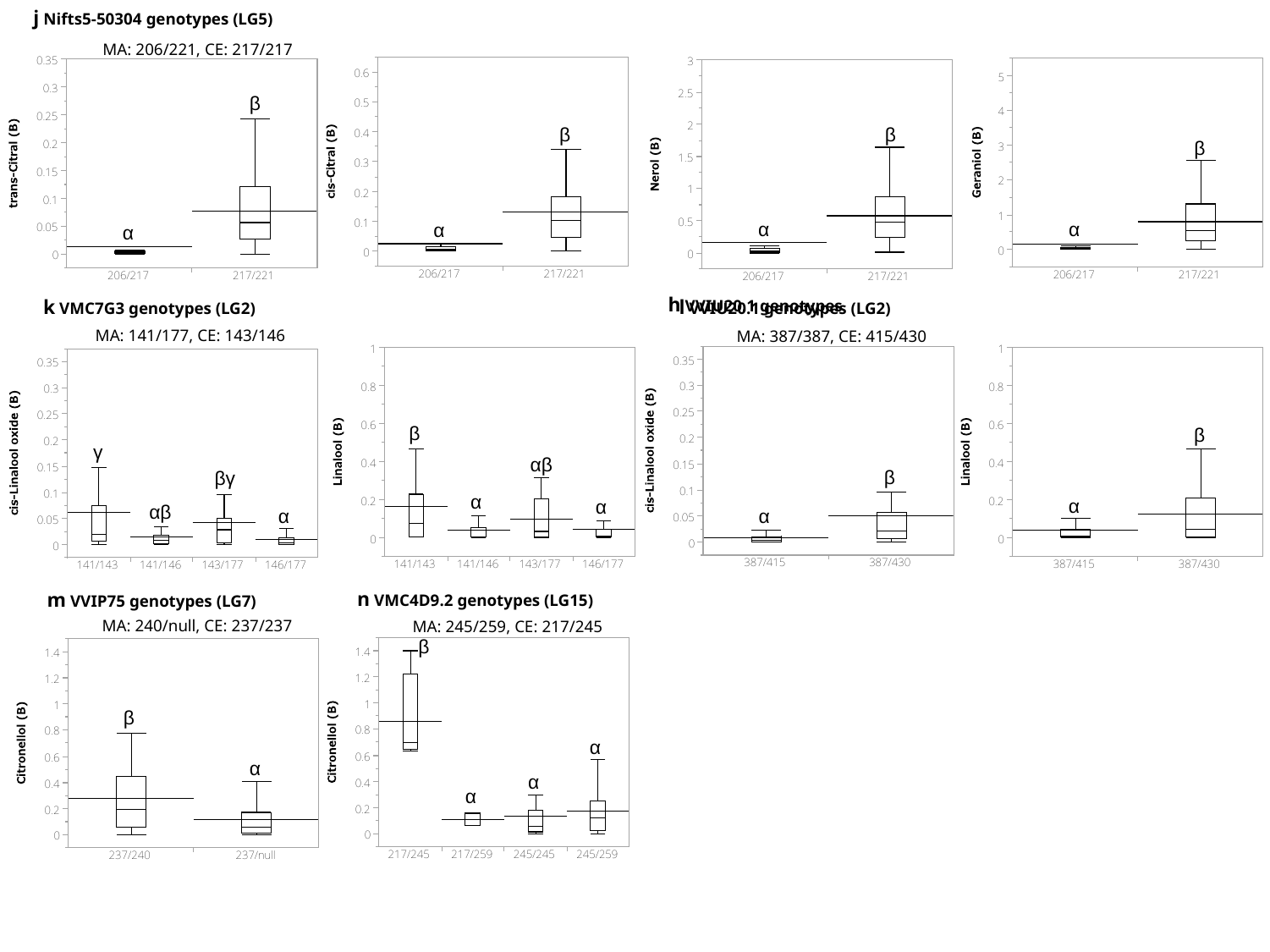

j Nifts5-50304 genotypes (LG5)
MA: 206/221, CE: 217/217
β
β
β
β
α
α
α
α
h VVIU20.1 genotypes
k VMC7G3 genotypes (LG2)
l VVIU20.1 genotypes (LG2)
MA: 141/177, CE: 143/146
MA: 387/387, CE: 415/430
β
β
γ
αβ
β
βγ
α
α
α
αβ
α
α
n VMC4D9.2 genotypes (LG15)
m VVIP75 genotypes (LG7)
MA: 240/null, CE: 237/237
MA: 245/259, CE: 217/245
β
β
α
α
α
α

## Slide 7
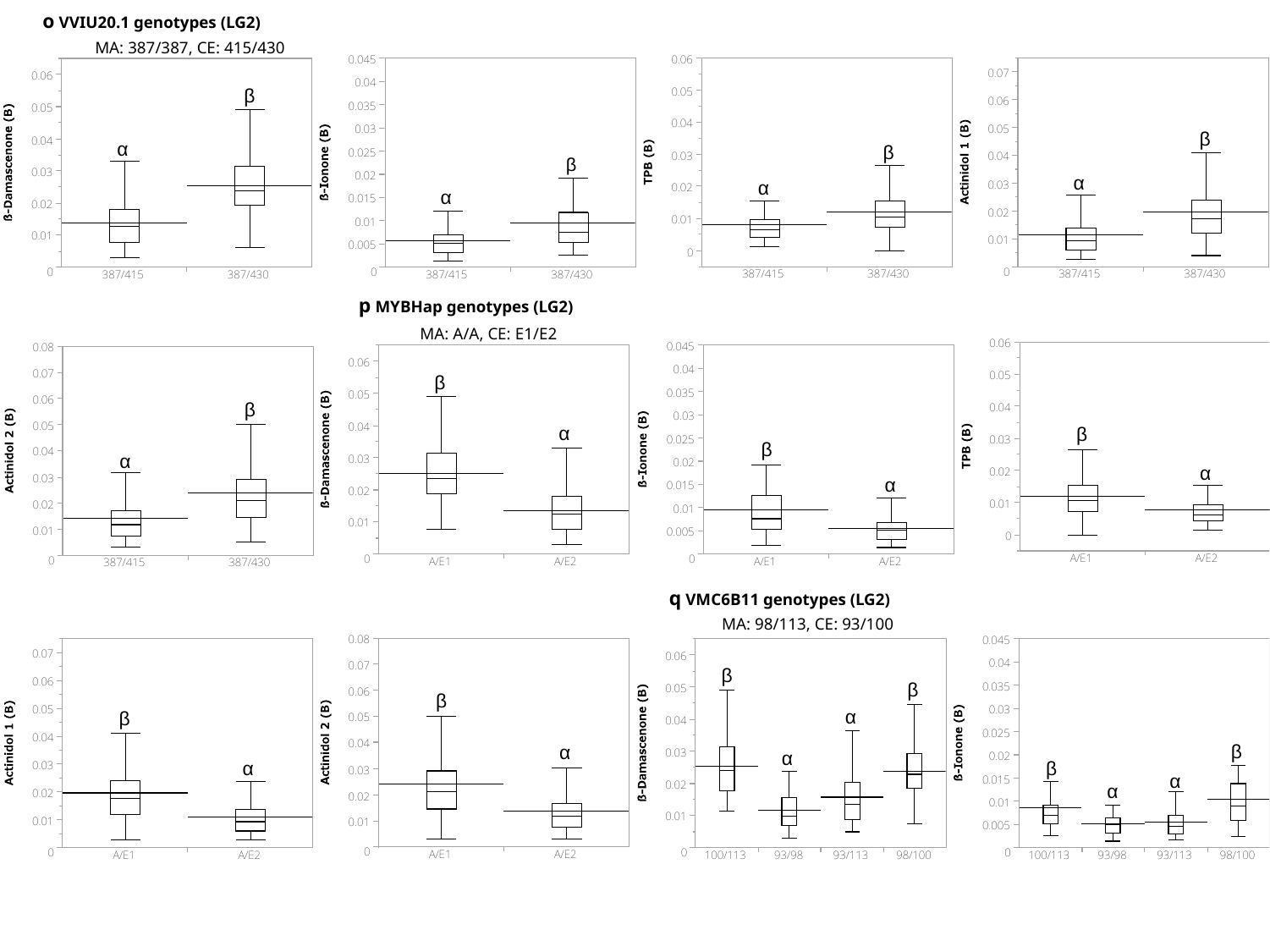

o VVIU20.1 genotypes (LG2)
MA: 387/387, CE: 415/430
β
β
α
β
β
α
α
α
p MYBHap genotypes (LG2)
MA: A/A, CE: E1/E2
β
β
α
β
β
α
α
α
q VMC6B11 genotypes (LG2)
MA: 98/113, CE: 93/100
β
β
β
α
β
β
α
α
β
α
α
α

## Slide 8
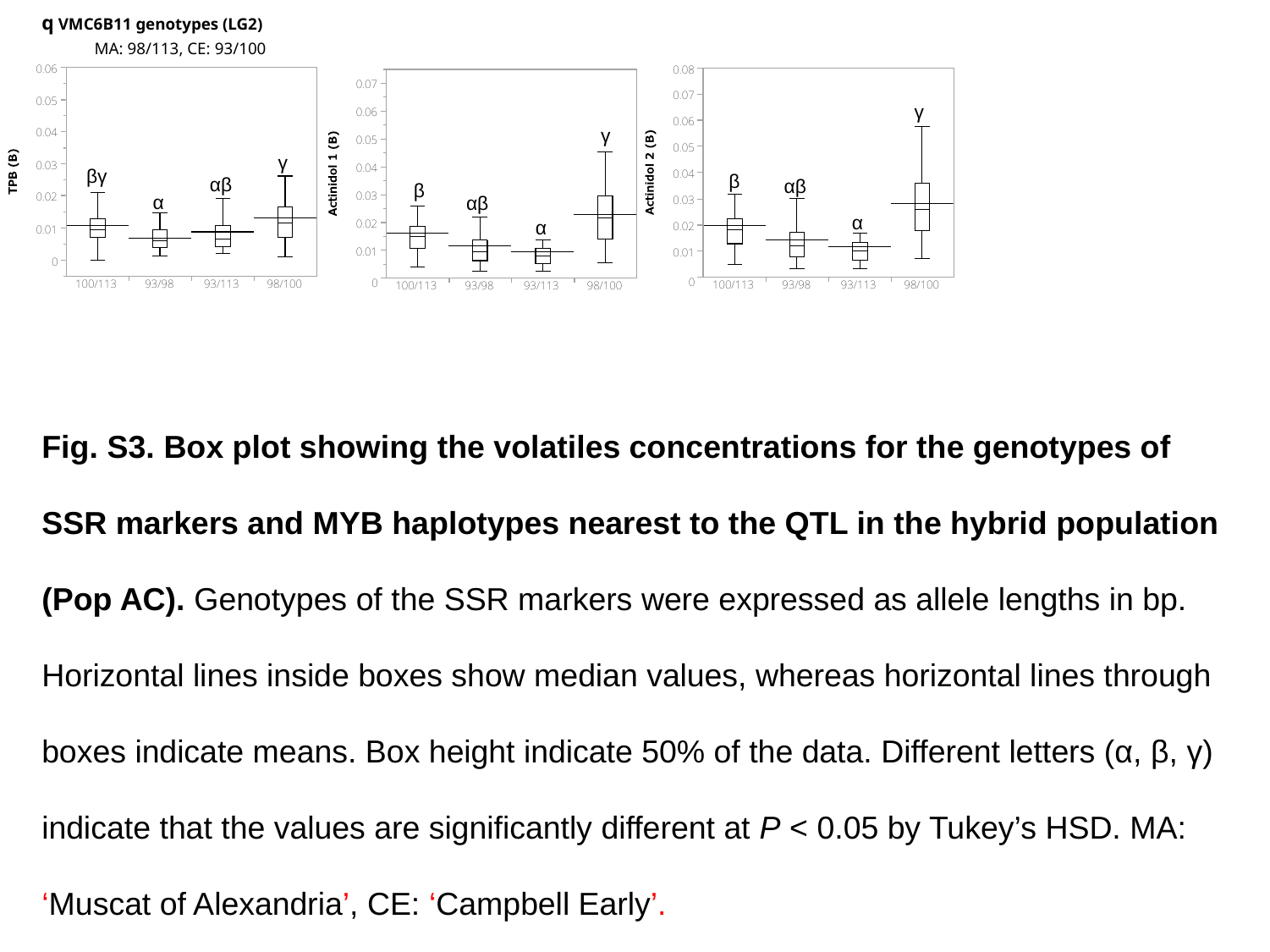

q VMC6B11 genotypes (LG2)
MA: 98/113, CE: 93/100
γ
γ
γ
βγ
β
αβ
αβ
β
α
αβ
α
α
Fig. S3. Box plot showing the volatiles concentrations for the genotypes of SSR markers and MYB haplotypes nearest to the QTL in the hybrid population (Pop AC). Genotypes of the SSR markers were expressed as allele lengths in bp. Horizontal lines inside boxes show median values, whereas horizontal lines through boxes indicate means. Box height indicate 50% of the data. Different letters (α, β, γ) indicate that the values are significantly different at P < 0.05 by Tukey’s HSD. MA: ‘Muscat of Alexandria’, CE: ‘Campbell Early’.
