# Supplementary material for: Genetic architecture of berry aroma compounds in a QTL (quantitative trait loci) mapping population of interspecific hybrid grapes (Vitis labruscana × Vitis vinifera)
Source: BMC Plant Biol. 2022 Sep 23;22:458. doi: 10.1186/s12870-022-03842-z (PMC9503205; doi:10.1186/s12870-022-03842-z)
Supplement: Supplementary file 4 — Additional file 4: Table S1. Free and bound volatile components identified in ‘Muscat of Alexandria’ and ‘Campbell Early’ berries. Table S2. Meteorological conditions in the Higashihiroshima region during the developmental period of grape berries in 2014 and 2015. Table S5. QTL analysis results showing the hybrid population’s volatile concentrations (Pop AC). Table S6. Newly designed primers used in this study. [file 12870_2022_3842_MOESM4_ESM.docx]

**Supplementary Tables**

## Table S1. Free and bound volatile components identified in ‘Muscat of Alexandria’ and ‘Campbell Early’ berries

| **Chemical group/** | **RI^b^** | **Identification** | **CAS number** | **Source,** |
| --- | --- | --- | --- | --- |
| **Compound name^a^** |  | **Assignment^c^** |  | **Purity** |
| **Lipid derivatives** |  |  |  |  |
| ***C_6_ compounds*** |  |  |  |  |
| 1-Hexanal (B) | 1098 | A | 000066-25-1 | Wako, 95% |
| 1-Hexanol (B) | 1371 | A | 000111-27-3 | TCI, 98% |
| ***Lactones*** |  |  |  |  |
| Furfural (B) | 1479 | A | 000098-01-1 | TCI, 98% |
| 5-Methyl furfural (B) | 1583 | A | 000620-02-0 | TCI, 97% |
| 4-methoxy-2,5-dimethyl 3(2H)-Furanone/Mesifurane (B, F) | 1603 | A | 003658-77-3 | TCI, 97% |
| *γ*-Nonalactone (B, F) | 2036 | A | 000104-61-0 | TCI, 98% |
| *γ*-Decalactone (B, F) | 2151 | B | 000706-14-9 | tentatively identified |
| ***Alcohols and aldehydes*** |  |  |  |  |
| Heptanal (B) | 1206 | B | 000111-71-7 | tentatively identified |
| 2-Hexenal (B, F) | 1208 | A | 000505-57-7 | Wako, 97% |
| 3-Methyl-1-butanol (B) | 1229 | A | 000123-92-2 | TCI, 90% |
| Octanal (B) | 1301 | A | 000124-13-0 | Wako, 97% |
| 1-Hepten-3-one (B) | 1316 | B | 002918-13-0 | tentatively identified |
| 2-Heptanol (B) | 1336 | A | 000543-49-7 | TCI, 98% |
| Nonanal (B) | 1402 | A | 000124-19-6 | Wako, 95% |
| 1-Octen-3-ol (B) | 1462 | A | 003391-86-4 | TCI, 98% |
| 2-Ethyl-1-hexanol (B, F) | 1505 | A | 000104-76-7 | TCI, 99% |
| 1-Octanol (B, F) | 1570 | A | 000111-87-5 | TCI, 99% |
| 1-Nonanol (B, F) | 1670 | A | 000143-08-8 | TCI, 97% |
| cis-3-Nonen-1-ol (B, F) | 1694 | A | 010340-23-5 | TCI, 99% |
| ***Esters*** |  |  |  |  |
| Hexyl acetate (B) | 1287 | A | 000142-92-7 | Wako, 97% |
| Heptyl acetate (B) | 1383 | A | 000112-06-1 | TCI, 99% |
| Ethyl nonanoate (B) | 1542 | B | 000123-29-5 | tentatively identified |
| **Shikimic acid derivatives** |  |  |  |  |
| ***Benzenes*** |  |  |  |  |
| Benzaldehyde (B, F) | 1534 | A | 000100-52-7 | Sigma Aldrich, 99% |
| 4-Methyl-benzaldehyde (B, F) | 1655 |  | 104-87-0 | Sigma Aldrich, 97% |
| Acetophenone (B, F) | 1658 | A | 000098-86-2 | TCI, 98.5% |
| Methyl salicylate (B) | 1783 | A | 000119-36-8 | TCI, 99% |
| Ethyl salicylate (B) | 1817 | A | 000118-61-6 | Wako, 98% |
| 3,4-Dimethyl benzaldehyde (B, F) | 1819 | A | 005779-95-3 | TCI, 95% |
| 2-Phenylethyl acetate (B, F) | 1823 | A | 000103-45-7 | TCI, 98% |
| Benzyl alcohol (B) | 1888 | B | 000100-51-6 | tentatively identified |
| Phenylethyl alcohol (B, F) | 1924 | A | 000060-12-8 | TCI, 98% |
| Methyl anthranilate (F) | 2267 | A | 000134-20-3 | TCI, 99% |
| *o*-Aminoacetophenone (F) |  | A | 000551-93-9 | TCI, 98% |
| 2,3-Dihydro-benzofuran (B) | >2500 | B | 496-16-2 | tentatively identified |
| Benzyl salicylate (B) | >2500 | A | 000118-58-1 | TCI, 99% |
| ***Volatile phenols*** |  |  |  |  |
| *o*-Guiacol (B) | 1869 | A | 000090-05-1 | Wako, 99% |
| Eugenol (B, F) | 2177 | A | 000097-53-0 | Wako, 95% |
| *o*-Thymol (B, F) | 2195 | A | 499-75-2 | TCI, 98% |
| 4-Vinylguaiacoｌ (B) | 2205 | A | 007786-61-0 | Penta Manufacturing Co., 10% |
| *p*-Thymol (B) | 2211 | A | 003228-02-2 | TCI, 99% |
| ***Vanillins*** |  |  |  |  |
| Vanillyl acetone/Zingerone (B) | >2500 | A | 000122-48-5 | TCI, 98% |
| Methyl vanillate (B, F) | >2500 | A | 003943-74-6 | TCI, 98% |
| **Terpenoids** |  |  |  |  |
| D-Limonene (B) | 1206 | A | 005989-27-5 | TCI, 95% |
| 1,8-Cineol (F) | 1220 | A | 000470-82-6 | TCI,99% |
| *γ*-Terpinene (B) | 1254 | A | 000099-85-4 | TCI,95% |
| cis-*β*-Ocimene (B) | 1262 | C | 003338-55-4 | tentatively identified |
| *o*-Cymene (B) | 1282 | A | 000527-84-4 | TCI, 99% |
| Terpinolene (B) | 1290 | A | 000586-62-9 | TCI, 85% |
| cis-Linalool oxide (B) | 1452 | A | 1000121-97-4 | TCI, 98% |
| Nerol oxide (B) | 1478 | B | 001786-08-9 | tentatively identified |
| Linalool (B, F) | 1556 | A | 000078-70-6 | TCI, 96% |
| Terpinen-4-ol (B) | 1607 | A | 000562-74-3 | TCI, 95% |
| cis-Citral (B, F) | 1686 | A | 000106-26-3 | Wako, 95% |
| *α*-Terpineol (B, F) | 1702 | A | 010482-56-1 | TCI, 95% |
| trans-Citral (B, F) | 1736 | A | 000141-27-5 | Wako, 95% |
| Citronellol (B, F) | 1777 | A | 000106-22-9 | Wako, 85% |
| Nerol (B, F) | 1811 | A | 000106-25-2 | TCI, 98% |
| Geranyl acetone (B) | 1856 | A | 000689-67-8 | TCI, 98% |
| Geraniol (B, F) | 1858 | A | 000106-24-1 | TCI, 96% |
| *δ*-Cadinene (B) | 2069 | C | 000483-76-1 | tentatively identified |
| Cedrol (B) | 2122 | C | 000077-53-2 | tentatively identified |
| *δ*-Cadinol (B) | 2155 | C | 36564-42-8 | tentatively identified |
| **Norisoprenoids** |  |  |  |  |
| Vitispirane A+B (B) | 1530 | B |  | tentatively identified |
| TDN (B) | 1748 | A |  | provided by Kirin Co. Ltd., 95% |
| *β*-Damascenone (B) | 1823 | A | 23726-93-4 | Sigma Aldrich, 95% |
| TPB (B) | 1833 | B | 1000357-25-7 | tentatively identified |
| Actinidol 1 (B) | 1921 | B |  | tentatively identified |
| Actinidol 2 (B) | 1935 | B |  | tentatively identified |
| *β*-ionone (B) | 1944 | A | 79-77-6 | Wako, 95% |

## ^a^The bound volatile compounds identified are indicated by (B), whereas the free volatile compounds identified are indicated by (F).

## ^b^RI = retention index on an HP-INNOWax column.

## ^c^The reliability of the proposed identification is indicated as follows:

## A- Identification carried out by comparing the gas chromatographic retention with mass spectrometric data of the pure and authentic reference compounds (pure compound available).

## B- Identification tentatively carried out by comparing the gas chromatographic retention with mass spectrometric data reported in the literature (pure compound not available).

## C- Pure reference compounds not available and there are no published values. Tentatively identified based on the RI and the coincidence of mass spectrometric data with the NIST Library 11.

## TDN (1,1,6,-trimethyl-1,2-dihydronapthalene); TPB (4-(2,3,6-trimethylphenyl)buta-1,3-diene); TCI- Tokyo Chemical Industry, Co. LTD, Japan.

## Table S2. Meteorological conditions in the Higashihiroshima region during the developmental period of grape berries in 2014 and 2015

|  | **Temperature (°C)  Average** | |  | **Rainfall (mm)  Total** | |  | **Sunshine duration (h) Total** | |
| --- | --- | --- | --- | --- | --- | --- | --- | --- |
| **month** | **2014^a^** | **2015** |  | **2014** | **2015** |  | **2014** | **2015** |
| April | 12.6 | 13.8 |  | 48.5 | 113 |  | 195.1 | 154.3 |
| May | 17.1 | 17.7 |  | 50.5 | 93.5 |  | 275.7 | 231 |
| June | 21 | 20 |  | 114 | 168.5 |  | 150 | 143.3 |
| July | 24.2 | 23.8 |  | 210.5 | 90 |  | 192 | 165.8 |
| August | 25.6 | 25.3 |  | 287 | 199 |  | 109.4 | 204.4 |
| September | 23 | 21.9 |  | 34.5 | 127.5 |  | 177.5 | 153.1 |

## ^a^ Year.

## Table S5. QTL analysis results showing the hybrid population’s volatile concentrations (Pop AC)

| **Volatile^a^** | **Chemical group** | **Year** | ***n*^b^** | **Linkage group (LG)** | **Map^c^** | **LOD peak** | **LOD threshold**  **α = 0.05** | **% variance explained** | **QTL position (cM)** | **Confidence interval 1.0-LOD (cM)** | **Nearest marker^d^** |
| --- | --- | --- | --- | --- | --- | --- | --- | --- | --- | --- | --- |
| Mesifurane (E) | Lactones | Mean | 81 | 6 | MA | 2.64 | 2.6 | 13.9 | 61.5 | 52.4-79.0 | GF06-11 |
|  |  | 2014 | 82 | 12 | CE | 3.36 | 2.7 | 17.2 | 11.7 | 7.6-20.8 | VMC5H4 |
|  |  | Mean | 81 | 12 | CE | 3.86 | 2.8 | 19.7 | 11.7 | 7.6-18.8 | VMC5H4 |
| Mesifurane (F) | Lactones | 2015 | 90 | 11 | CE | 3.17 | 2.7 | 15.1 | 27.0 | 21.6-31.2 | Nifts11-7343 |
|  |  | 2015 | 90 | 12 | CE | 4.51 | 2.7 | 20.8 | 11.6 | 7.6-17.8 | VMC5H4 |
|  |  | Mean | 81 | 11 | CE | 2.63 | 2.6 | 13.9 | 24.5 | 21.6-24.8 | Nifts11-7518 |
|  |  | Mean | 81 | 12 | CE | 4.05 | 2.6 | 20.6 | 11.7 | 6.6-19.8 | VMC5H4 |
|  |  | 2015 | 90 | 12 | Consensus | 5.03 | 4.9 | 22.9 | 10.8 | 5.9-18.9 | VMC5H4 |
|  |  | Mean | 81 | 12 | Consensus | 5.14 | 4.8 | 25.4 | 14.9 | 7.9-21.9 | VMC5H4 |
| γ-Decalactone (B) | Lactones | 2015 | 90 | 13 | MA | 4.26 | 2.7 | 19.6 | 26.8 | 7.3-39.8 | Nifts13-13599 |
|  |  | 2015 | 90 | 9 | MA | 2.73 | 2.7 | 13 | 57.6 | 50.0-Bottom | VMC3H5 |
|  |  | Mean | 81 | 2 | MA | 2.84 | 2.7 | 14.9 | 0 | 0.0-10.0 | UDV109 |
|  |  | Mean | 81 | 13 | MA | 2.72 | 2.7 | 14.3 | 28.8 | 5.3-46.2 | Nifts13-13599 |
|  |  | 2015 | 90 | 8 | CE | 4.14 | 2.8 | 19.1 | 87 | 82.1-94.0 | VVIB66 |
|  |  | 2015 | 90 | 8 | Consensus | 4.92 | 4.5 | 22.2 | 63 | 58.1-70.0 | VVIB66 |
| 1-Octanol (B) | Alcohols and aldehydes | 2015 | 90 | 8 | CE | 3.02 | 2.8 | 14.3 | 91 | 65.1-101.8 | VVIB66 |
| cis-3-Nonen-1-ol (B) | Alcohols and aldehydes | 2015 | 90 | 8 | CE | 3.39 | 2.6 | 15.9 | 89 | 66.1-101.8 | VVIB66 |
| cis-3-Nonen-1-ol (F) | Alcohols and aldehydes | 2014 | 82 | 8 | CE | 2.98 | 2.8 | 15.2 | 70.1 | 54.2-97.8 | VMC5H2 |
|  |  | 2015 | 90 | 8 | CE | 2.91 | 2.6 | 14 | 62.8 | 56.2-76.1 | VMC5H2 |
|  |  | Mean | 81 | 8 | CE | 3.2 | 2.7 | 16.6 | 62.8 | 55.2-78.1 | VMC5H2 |
| Phenetyl acetate (B) | Benzenes | 2014 | 82 | 17 | MA | 2.99 | 2.6 | 15.5 | 62 | 55.2-Bottom | GF17-07 |
|  |  | 2014 | 82 | 17 | Consensus | 5.12 | 5 | 25 | 50.2 | 46.2-56.8 | VVIB09 |
| Phenetyl acetate (F) | Benzenes | Mean | 81 | 11 | MA | 3.07 | 2.8 | 16 | 57.8 | 53.3-58.7 | GF11-10 |
| Phenetyl alcohol (B) | Benzenes | 2014 | 82 | 7 | MA | 5.8 | 3 | 27.8 | 80.1 | 66.1-86.9 | VMC1A12 |
|  |  | 2015 | 90 | 7 | MA | 6.14 | 2.9 | 27 | 67.1 | 55.1-80.1 | VMC1A12 |
|  |  | Mean | 81 | 7 | MA | 7.07 | 2.9 | 33.1 | 77.1 | 66.1-86.3 | VMC1A12 |
|  |  | Mean | 81 | 2 | CE | 3.15 | 3.1 | 16.4 | 27.8 | 17.7-35.3 | VVS3 |
|  |  | 2014 | 82 | 7 | Consensus | 6.77 | 4.5 | 31.6 | 61.8 | 53.8-64.4 | VMC1A12 |
|  |  | 2015 | 90 | 7 | Consensus | 6.48 | 4.2 | 28.2 | 50.8 | 44.4-59.8 | VMC8D11 |
|  |  | 2015 | 90 | 16 | Consensus | 5.16 | 4.2 | 23.2 | 51.3 | 48.9-Bottom | Nifts16-28513 |
|  |  | Mean | 81 | 7 | Consensus | 7.54 | 4.5 | 34.9 | 59.8 | 52.8-64.4 | VMC1A12 |
| Methyl anthranilate (F) | Benzenes | 2015 | 90 | 7 | MA | 1.77 | 1.4 | 8.6 | 17.1 | 0.0-53.1 | UDV011 |
| o-Aminoacetophenone (F) | Benzenes | 2014 | 82 | 3 | MA | 3.18 | 2.5 | 16 | 34.9 | 21.8-40.0 | VVIN54 |
|  |  | 2015 | 90 | 7 | MA | 2.66 | 2.4 | 12.7 | 31.7 | 15.1-39.8 | VVIS58 |
|  |  | Mean | 81 | 3 | MA | 2.74 | 2.7 | 14.1 | 34.9 | 22.8-42.0 | VVIN54 |
|  |  | 2014 | 82 | 3 | CE | 3.28 | 2.7 | 16.5 | 16.1 | 6.3-29.1 | UDV021 |
|  |  | Mean | 81 | 3 | CE | 3.35 | 2.6 | 16.9 | 18.1 | 6.5-30.1 | UDV021 |
|  |  | 2014 | 82 | 3 | Consensus | 5.41 | 5.1 | 25.7 | 23 | 15.0-29.0 | VMC1A5 |
|  |  | Mean | 81 | 3 | Consensus | 5.99 | 4.7 | 28.3 | 22 | 16.0-29.0 | VMC1A5 |
| Eugenol (B) | Volatile phenols | 2015 | 90 | 14 | CE | 3.17 | 3 | 15 | 40.3 | 27.8-46.3 | VVIP22 |
| Eugenol (F) | Volatile phenols | 2015 | 90 | 7 | CE | 2.67 | 2.3 | 12.9 | 49.2 | 35.7-70.4 | GF07-04 |
|  |  | Mean | 81 | 7 | CE | 3.21 | 2.6 | 16.7 | 50.2 | 39.7-66.2 | GF07-04 |
|  |  | 2014 | 82 | 7 | Consensus | 5.12 | 4.7 | 24.7 | 68.1 | 52.8-79.6 | GF07-13 |
| Methyl vanillate (B) | Vanillins | 2014 | 82 | 6 | MA | 2.85 | 2.5 | 14.8 | 46.9 | 36.9-53.4 | GF06-08 |
| 1,8-Cineol (F) | Terpenoids | 2014 | 82 | 13 | CE | 3.86 | 2.5 | 19.3 | 48.2 | 32.6-56.0 | VMC9H4.2 |
|  |  | 2015 | 90 | 13 | CE | 5.88 | 2.6 | 26.2 | 41.5 | 34.6-49.2 | VMC9H4.2 |
|  |  | Mean | 81 | 13 | CE | 5.4 | 2.5 | 26.4 | 41.5 | 35.4-52.2 | VMC9H4.2 |
|  |  | 2015 | 90 | 13 | Consensus | 5.86 | 4.9 | 26.1 | 48.3 | 35.0-56.4 | VMC9H4.2 |
|  |  | Mean | 81 | 13 | Consensus | 5.49 | 5 | 26.8 | 48.3 | 36.0-58.4 | VMC9H4.2 |
| γ-Terpinene (B) | Terpenoids | 2015 | 90 | 17 | MA | 2.95 | 2.5 | 14 | 24.6 | 14-43.4 | VVIQ22.2 |
|  |  | 2015 | 90 | 5^e^ | MA | 2.88 | 2.5 | 13.7 | 6.8 | 0.0-31.4 | Nifts5-50958 |
|  |  | 2014 | 82 | 13 | CE | 2.78 | 2.5 | 14.5 | 48.2 | 30.6-57.4 | VMC9H4.2 |
|  |  | 2015 | 90 | 13 | CE | 4.42 | 2.6 | 20.2 | 48.2 | 38.5-56.0 | VMC9H4.2 |
|  |  | Mean | 81 | 13 | CE | 3.5 | 2.5 | 18.1 | 48.2 | 36.5-Bottom | VMC9H4.2 |
|  |  | 2014 | 82 | 13 | Consensus | 5.28 | 4.8 | 25.7 | 41.3 | 36.0-47.3 | VVIC51 |
|  |  | Mean | 81 | 13 | Consensus | 4.76 | 4.7 | 23.7 | 41.3 | 35.0-57.4 | VVIC51 |
| cis-Linalool oxide (B) | Terpenoids | 2014 | 82 | 2^f^ | CE | 3.57 | 2.5 | 18.2 | 59.4 | 43.4-Bottom | VMC7G3 |
|  |  | 2015 | 90 | 2^f^ | CE | 2.51 | 2.3 | 12.1 | 57.4 | 42.4-Bottom | VVIU20.1 |
|  |  | 2015 | 90 | 3 | CE | 2.64 | 2.3 | 12.6 | 22.1 | 10.5-37.6 | UDV021 |
|  |  | Mean | 81 | 2^f^ | CE | 3.93 | 2.6 | 20 | 59.4 | 46.0-Bottom | VVIU20.1 |
| α-Terpineol (B) | Terpenoids | 2014 | 82 | 5^e^ | MA | 3.65 | 2.8 | 18.5 | 6.8 | 0.0-18.5 | Nifts5-50958 |
|  |  | 2015 | 90 | 5^e^ | MA | 9.42 | 2.7 | 38.3 | 2.3 | 0.0-12.3 | Nifts5-50304 |
|  |  | Mean | 81 | 5^e^ | MA | 5.88 | 2.9 | 28.4 | 6.8 | 1-12.3 | Nifts5-50958 |
|  |  | 2015 | 90 | 5^e^ | Consensus | 9.98 | 4.5 | 40 | 23.1 | 0.0-24.7 | Nifts5-50304 |
|  |  | Mean | 81 | 5^e^ | Consensus | 6.25 | 4.5 | 29.9 | 30 | 20.7-32.8 | Nifts5-51090 |
| α-Terpineol (F) | Terpenoids | 2014 | 82 | 5^e^ | MA | 3.44 | 2.8 | 17.4 | 7.7 | 5.6-13.3 | Nifts5-50937 |
|  |  | 2015 | 90 | 5^e^ | MA | 4.51 | 2.7 | 20.8 | 7.7 | 5.4-13.4 | Nifts5-50937 |
|  |  | Mean | 81 | 5^e^ | MA | 4.34 | 2.9 | 21.9 | 7.7 | 5.6-13.3 | Nifts5-50937 |
|  |  | 2015 | 90 | 5^e^ | Consensus | 6.1 | 4.5 | 27.1 | 30 | 27.3-41.5 | Nifts5-51090 |
|  |  | Mean | 81 | 5^e^ | Consensus | 5.81 | 4.6 | 28.1 | 30 | 27.3-41.5 | Nifts5-51090 |
| cis-Citral (B) | Terpenoids | 2014 | 82 | 5^e^ | MA | 6.66 | 2.8 | 31.2 | 7.7 | 4.4-11.7 | Nifts5-50937 |
|  |  | 2015 | 90 | 5^e^ | MA | 9.05 | 2.9 | 37.1 | 7.7 | 3.3-12.3 | Nifts5-50937 |
|  |  | Mean | 81 | 5^e^ | MA | 7.82 | 2.7 | 35.9 | 7.7 | 4.4-10.7 | Nifts5-50937 |
|  |  | 2014 | 82 | 5^e^ | Consensus | 7.59 | 4.6 | 34.7 | 30 | 26.3-32.0 | Nifts5-51090 |
|  |  | 2015 | 90 | 5^e^ | Consensus | 9.36 | 4.6 | 38.1 | 27.3 | 24.0-32.8 | Nifts5-50852 |
|  |  | Mean | 81 | 5^e^ | Consensus | 8.51 | 4.8 | 38.4 | 30 | 26.3-32.0 | Nifts5-51090 |
| cis-Citral (F) | Terpenoids | 2014 | 82 | 5^e^ | MA | 3.41 | 2.8 | 17.2 | 4.4 | 0.0-13.3 | Nifts5-50665 |
|  |  | 2015 | 90 | 5^e^ | MA | 3.08 | 2.6 | 14.7 | 10.7 | 3.3-20.5 | Nifts5-51172 |
|  |  | Mean | 81 | 5^e^ | MA | 4.02 | 2.8 | 20.4 | 10.7 | 0.0-20.5 | Nifts5-51172 |
|  |  | Mean | 81 | 5^e^ | Consensus | 5.09 | 4.6 | 25.1 | 27.3 | 22.4-30.0 | Nifts5-50852 |
| trans-Citral (B) | Terpenoids | 2014 | 82 | 5^e^ | MA | 4.77 | 2.8 | 23.5 | 6.8 | 0.0-12.3 | Nifts5-50958 |
|  |  | 2015 | 90 | 5^e^ | MA | 9.72 | 2.8 | 39.2 | 6.8 | 3.3-11.7 | Nifts5-50958 |
|  |  | Mean | 81 | 5^e^ | MA | 6.58 | 2.8 | 31.2 | 6.8 | 1.3-11.7 | Nifts5-50958 |
|  |  | 2014 | 82 | 5^e^ | Consensus | 5.22 | 4.6 | 25.4 | 30 | 20.7-35.0 | Nifts5-51090 |
|  |  | 2015 | 90 | 5^e^ | Consensus | 11.14 | 4.5 | 43.5 | 27.3 | 26.3-27.6 | Nifts5-50852 |
|  |  | 2015 | 90 | 7 | Consensus | 5.08 | 4.5 | 22.9 | 19.5 | 11.4-25.4 | UDV011 |
|  |  | Mean | 81 | 5^e^ | Consensus | 7.08 | 4.6 | 33.1 | 27.3 | 21.9-32.0 | Nifts5-50852 |
| trans-Citral (F) | Terpenoids | 2014 | 82 | 5^e^ | MA | 3.7 | 2.8 | 18.6 | 5.4 | 4.4-13.3 | Nifts5-50958 |
|  |  | 2015 | 90 | 5^e^ | MA | 3.7 | 2 | 17.4 | 6.8 | 0.0-21.5 | Nifts5-50958 |
|  |  | Mean | 81 | 5^e^ | MA | 4.99 | 2.8 | 24.7 | 6.8 | 4.4-13.3 | Nifts5-50958 |
|  |  | 2015 | 90 | 5^e^ | Consensus | 4.63 | 4.6 | 21.3 | 27.3 | 21.9-32.8 | Nifts5-50958 |
|  |  | Mean | 81 | 5^e^ | Consensus | 5.2 | 4.5 | 25.6 | 30 | 25.1-33.8 | Nifts5-51090 |
| Citronellol (B) | Terpenoids | 2014 | 82 | 5^e^ | MA | 7.15 | 2.8 | 33.1 | 7.7 | 5.6-12.3 | Nifts5-50937 |
|  |  | 2014 | 82 | 7 | MA | 3.11 | 2.8 | 16 | 92.1 | 60.1-92.1 | VVIP75 |
|  |  | 2015 | 90 | 5^e^ | MA | 6.8 | 2.7 | 29.4 | 7.7 | 5.6-12.3 | Nifts5-50937 |
|  |  | 2015 | 90 | 7 | MA | 2.88 | 2.7 | 13.7 | 92.6 | 54.1-106.3 | VVIP75 |
|  |  | Mean | 81 | 5^e^ | MA | 7.47 | 2.7 | 34.6 | 7.7 | 6.8-11.7 | Nifts5-50937 |
|  |  | Mean | 81 | 7 | MA | 4.01 | 2.7 | 20.4 | 92.1 | 62.1-94.6 | VVIP75 |
|  |  | 2014 | 82 | 15 | CE | 3.26 | 2.8 | 16.7 | 31.5 | 26.5-33.0 | VMC4D9.2 |
|  |  | 2015 | 90 | 15 | CE | 3.76 | 2.8 | 17.5 | 33 | 29.5-Bottom | VMC4D9.2 |
|  |  | Mean | 81 | 15 | CE | 3.61 | 2.8 | 18.6 | 32.5 | 27.5-Bottom | VMC4D9.2 |
|  |  | 2014 | 82 | 5^e^ | Consensus | 7.86 | 4.5 | 35.7 | 29.5 | 27.3-32.0 | VVII52 |
|  |  | 2014 | 82 | 15 | Consensus | 5.85 | 4.5 | 28 | 31.8 | 24.4-Bottom | VMC8G3.2 |
|  |  | 2015 | 90 | 5^e^ | Consensus | 7.27 | 4.9 | 31.1 | 29.5 | 27.3-32.8 | VVII52 |
|  |  | 2015 | 90 | 15 | Consensus | 6.91 | 4.9 | 29.8 | 29.8 | 25.4-Bottom | VMC4D9.2 |
|  |  | Mean | 81 | 5^e^ | Consensus | 8.09 | 4.7 | 36.9 | 29.5 | 27.3-32.0 | VVII52 |
|  |  | Mean | 81 | 15 | Consensus | 6.74 | 4.7 | 31.8 | 30.8 | 24.4-Bottom | VMC4D9.2 |
| Citronellol (F) | Terpenoids | 2014 | 82 | 5^e^ | MA | 6.02 | 2.7 | 28.4 | 7.7 | 6.8-11.7 | Nifts5-50937 |
|  |  | 2015 | 90 | 5^e^ | MA | 5.32 | 2.2 | 24.1 | 7.7 | 6.8-14.4 | Nifts5-50937 |
|  |  | Mean | 81 | 5^e^ | MA | 6.15 | 2.4 | 29.5 | 7.7 | 6.8-12.3 | Nifts5-50937 |
|  |  | 2014 | 82 | 5^e^ | Consensus | 6.08 | 4.7 | 28.6 | 28.1 | 27.3-32.8 | Nifts5-50937 |
|  |  | 2015 | 90 | 5^e^ | Consensus | 5.83 | 4.6 | 26 | 29.5 | 27.3-32.8 | VVII52 |
|  |  | Mean | 81 | 5^e^ | Consensus | 6.36 | 4.6 | 30.3 | 29.5 | 27.3-32.8 | VVII52 |
| Linalool (B) | Terpenoids | 2014 | 82 | 5^e^ | MA | 3.05 | 2.4 | 15.7 | 4.3 | 0.0-19.5 | Nifts5-50665 |
|  |  | 2015 | 90 | 5^e^ | MA | 5.24 | 2.3 | 23.5 | 2.3 | 0.0-5.4 | Nifts5-50304 |
|  |  | Mean | 81 | 5^e^ | MA | 3.89 | 2.3 | 19.8 | 6.8 | 0.0-13.3 | Nifts5-50958 |
|  |  | Mean | 81 | 2^f^ | CE | 2.59 | 2.5 | 13.7 | 60.4 | 45.4-Bottom | VMC7G3 |
|  |  | 2015 | 90 | 5^e^ | Consensus | 5.61 | 4.7 | 24.9 | 30 | 26.1-32.8 | Nifts5-51090 |
| Linalool (F) | Terpenoids | 2014 | 82 | 5^e^ | MA | 3.33 | 2.5 | 16.9 | 7.7 | 5.4-21.5 | Nifts5-50937 |
|  |  | 2015 | 90 | 5^e^ | MA | 3.47 | 2.6 | 16.4 | 6.8 | 5.4-36.2 | Nifts5-50958 |
|  |  | Mean | 81 | 5^e^ | MA | 3.67 | 2.8 | 18.8 | 7.7 | 5.4-34.4 | Nifts5-50937 |
| Nerol (B) | Terpenoids | 2014 | 82 | 5^e^ | MA | 4.32 | 2.8 | 21.6 | 7.7 | 3.3-13.3 | Nifts5-50937 |
|  |  | 2015 | 90 | 5^e^ | MA | 12.55 | 2.9 | 47.4 | 7.7 | 5.6-11.7 | Nifts5-50937 |
|  |  | Mean | 81 | 5^e^ | MA | 7.26 | 3.1 | 33.8 | 7.7 | 4.4-11.7 | Nifts5-50937 |
|  |  | 2014 | 82 | 5^e^ | Consensus | 4.95 | 4.7 | 24.3 | 30 | 24.0-32.8 | Nifts5-51090 |
|  |  | 2015 | 90 | 5^e^ | Consensus | 12.8 | 4.4 | 48.1 | 30 | 28.6-32.0 | Nifts5-51090 |
|  |  | Mean | 81 | 5^e^ | Consensus | 7.96 | 4.6 | 36.4 | 30 | 26.1-32.0 | Nifts5-51090 |
| Nerol (F) | Terpenoids | 2014 | 82 | 5^e^ | MA | 6.82 | 2.8 | 31.5 | 6.8 | 1.3-11.7 | Nifts5-50958 |
|  |  | 2015 | 90 | 5^e^ | MA | 7.45 | 2.7 | 32 | 7.7 | 4.4-13.3 | Nifts5-50937 |
|  |  | Mean | 81 | 5^e^ | MA | 7.14 | 2.8 | 33.4 | 6.8 | 3.3-11.7 | Nifts5-50958 |
|  |  | 2014 | 82 | 5^e^ | Consensus | 7.53 | 4.7 | 34.2 | 27.3 | 24.0-27.6 | Nifts5-50958 |
|  |  | 2015 | 90 | 5^e^ | Consensus | 8.07 | 4.8 | 34.1 | 27.3 | 25.1-32.0 | Nifts5-50852 |
|  |  | Mean | 81 | 5^e^ | Consensus | 7.94 | 4.6 | 36.3 | 27.3 | 25.1-27.6 | Nifts5-50852 |
| Geraniol (B) | Terpenoids | 2014 | 82 | 5^e^ | MA | 3.86 | 2.7 | 19.5 | 6.8 | 3.3-12.3 | Nifts5-50958 |
|  |  | 2015 | 90 | 5^e^ | MA | 10.07 | 2.9 | 40.3 | 2.3 | 0.0-11.7 | Nifts5-50304 |
|  |  | Mean | 81 | 5^e^ | MA | 5.97 | 2.9 | 28.8 | 6.8 | 3.3-11.7 | Nifts5-50958 |
|  |  | 2015 | 90 | 3 | CE | 2.84 | 2.8 | 13.5 | 36.6 | 14.1-42.6 | VVMD36 |
|  |  | 2015 | 90 | 5^e^ | Consensus | 11.69 | 4.5 | 45 | 27.3 | 26.3-27.6 | Nifts5-50852 |
|  |  | 2015 | 90 | 7 | Consensus | 5.15 | 4.5 | 23.2 | 19.5 | 11.4-25.4 | UDV011 |
|  |  | Mean | 81 | 5^e^ | Consensus | 6.73 | 4.5 | 31.8 | 27.3 | 24.0-27.6 | Nifts5-50958 |
| Geraniol (F) | Terpenoids | 2014 | 82 | 5^e^ | MA | 5.42 | 2.7 | 26 | 6.8 | 0.0-11.7 | Nifts5-50958 |
|  |  | 2015 | 90 | 5^e^ | MA | 4.25 | 2.1 | 19.7 | 6.8 | 3.3-20.5 | Nifts5-50958 |
|  |  | 2015 | 90 | 7 | MA | 2.34 | 2.1 | 11.4 | 58.1 | 39.8-81.1 | Nifts7-59300 |
|  |  | Mean | 81 | 5^e^ | MA | 5.46 | 2.6 | 26.7 | 6.8 | 3.3-12.3 | Nifts5-50958 |
|  |  | 2014 | 82 | 5^e^ | Consensus | 7.09 | 4.3 | 32.5 | 27.3 | 25.1-28.6 | Nifts5-50958 |
|  |  | 2015 | 90 | 5^e^ | Consensus | 4.93 | 4.9 | 22.5 | 27.3 | 25.1-32.8 | Nifts5-50958 |
|  |  | Mean | 81 | 5^e^ | Consensus | 6.74 | 4.9 | 31.8 | 27.3 | 25.1-28.6 | Nifts5-50958 |
| TDN (B) | Norisoprenoids | 2015 | 90 | 10 | MA | 2.92 | 2.9 | 13.9 | 0 | 0.0-11.5 | Nifts10-3733 |
|  |  | Mean | 81 | 10 | MA | 3.27 | 2.5 | 18 | 31.3 | 23.4-38.9 | Nifts10-3949 |
|  |  | 2015 | 90 | 2 | CE | 3.62 | 2.8 | 16.9 | 21.6 | 17.7-45.4 | Nifts2-41040 |
| ß-Damascenone (B) | Norisoprenoids | 2014 | 82 | 2 | CE | 8.43 | 2.8 | 39.6 | 54 | 47.0-57.4 | VVIU20.1 |
|  |  | 2015 | 90 | 2 | CE | 13.57 | 2.8 | 50.1 | 53 | 47.0-56.4 | MYB-Hap |
|  |  | Mean | 81 | 2 | CE | 13.22 | 2.8 | 55.1 | 54 | 52.0-56.4 | VVIU20.1 |
|  |  | 2014 | 82 | 2 | Consensus | 9.03 | 4.3 | 41.7 | 48.4 | 44.2-55.4 | MYB-Hap |
|  |  | 2015 | 90 | 2 | Consensus | 13.78 | 4.5 | 50.6 | 52.1 | 46.4-55.4 | MYB-Hap |
|  |  | Mean | 81 | 2 | Consensus | 13.57 | 4.3 | 56.1 | 53.1 | 45.4-55.4 | VVIU20.1 |
| ß-Ionone (B) | Norisoprenoids | 2015 | 90 | 12 | MA | 3.24 | 2.3 | 15.3 | 8 | 0.0-20.8 | VMC5H4 |
|  |  | 2014 | 82 | 2 | CE | 2.93 | 2.5 | 16.1 | 48 | 46.0-60.4 | VMC6B11 |
|  |  | 2015 | 90 | 2 | CE | 5.12 | 2.4 | 23.1 | 42.4 | 34.3-54.0 | VMC6B11 |
|  |  | Mean | 81 | 2 | CE | 4.56 | 2.6 | 24.1 | 48 | 39.4-54.4 | VMC6B11 |
|  |  | 2015 | 90 | 2 | Consensus | 5.52 | 4.5 | 24.6 | 48.4 | 37.9-53.4 | MYB-Hap |
|  |  | Mean | 81 | 2 | Consensus | 6.08 | 4.6 | 30.8 | 48.4 | 40.9-53.4 | MYB-Hap |
| TPB (B) | Norisoprenoids | 2015 | 90 | 2 | CE | 6.17 | 2.9 | 27.1 | 41.4 | 34.3-46.0 | VMC2C10.1 |
|  |  | Mean | 81 | 2 | CE | 3.05 | 2.8 | 16.9 | 49 | 38.4-60.4 | MYB-Hap |
|  |  | 2015 | 90 | 2 | Consensus | 7.71 | 4.5 | 32.6 | 49.4 | 44.4-53.1 | MYB-Hap |
| Actinidol 1 (B) | Norisoprenoids | 2014 | 82 | 10 | MA | 3.75 | 2.8 | 20.1 | 28.7 | 25.4-31.3 | VRZAG64 |
|  |  | 2015 | 90 | 12 | MA | 3.2 | 2.4 | 15.1 | 11.1 | 0.0-21.8 | VMC8G6 |
|  |  | Mean | 81 | 10 | MA | 3.03 | 2.9 | 16.8 | 29 | 24.4-34.9 | Nifts10-3938 |
|  |  | 2014 | 82 | 2 | CE | 4.35 | 2.8 | 22.9 | 42.4 | 29.0-54.0 | VMC6B11 |
|  |  | 2015 | 90 | 2 | CE | 4.68 | 2.5 | 21.3 | 41.4 | 32.3-52.0 | VMC2C10.1 |
|  |  | Mean | 81 | 2 | CE | 5.17 | 2.9 | 26.9 | 41.4 | 30.0-52.0 | VMC2C10.1 |
|  |  | 2014 | 82 | 2 | Consensus | 6.97 | 4.6 | 34.1 | 48.4 | 40.9-53.1 | MYB-Hap |
|  |  | 2014 | 82 | 10 | Consensus | 5.33 | 4.6 | 27.3 | 36.2 | 35.7-38.3 | VRZAG64 |
|  |  | 2015 | 90 | 2 | Consensus | 5.21 | 4.7 | 23.4 | 41.9 | 35.4-56.4 | VMC5G7 |
|  |  | Mean | 81 | 2 | Consensus | 8.51 | 4.4 | 40.3 | 48.4 | 44.4-53.1 | MYB-Hap |
|  |  | Mean | 81 | 10 | Consensus | 4.4 | 4.4 | 23.4 | 36.2 | 35.7-38.3 | VRZAG64 |
| Actinidol 2 (B) | Norisoprenoids | 2014 | 82 | 10 | MA | 3.69 | 2.8 | 19.8 | 28.7 | 25.4-30.3 | VRZAG64 |
|  |  | 2015 | 90 | 12 | MA | 3.36 | 2.5 | 15.8 | 11.1 | 1.0-20.8 | VMC8G6 |
|  |  | Mean | 81 | 10 | MA | 3.02 | 2.8 | 16.7 | 29 | 24.4-34.9 | Nifts10-3938 |
|  |  | 2014 | 82 | 2 | CE | 4.38 | 2.9 | 23 | 41.4 | 29.0-53.0 | VMC2C10.1 |
|  |  | 2015 | 90 | 2 | CE | 4.92 | 2.7 | 22.2 | 41.4 | 32.3-52.0 | VMC2C10.1 |
|  |  | Mean | 81 | 2 | CE | 5.16 | 2.9 | 26.8 | 41.4 | 30.0-52.0 | VMC2C10.1 |
|  |  | 2014 | 82 | 2 | Consensus | 6.97 | 4.6 | 34.1 | 48.4 | 38.9-52.1 | MYB-Hap |
|  |  | 2014 | 82 | 10 | Consensus | 4.7 | 4.6 | 24.5 | 35.1 | 31.3-35.5 | Nifts10-3938 |
|  |  | 2015 | 90 | 12 | Consensus | 4.34 | 4.6 | 19.9 | 11.8 | 4.5-19.9 | VMC8G6 |
|  |  | Mean | 81 | 2 | Consensus | 8.45 | 4.6 | 40.1 | 48.4 | 44.4-53.1 | MYB-Hap |

**A full list showing all detected QTLs, including those only identified in a single year are shown. ^a^ Bound volatiles are indicated by (B), whereas free volatiles are indicated by (F). ^b^ Number of individuals used for QTL analysis. ^c^ MA: ‘Muscat of Alexandria’, CE: ‘Campbell Early’ ^d^ Marker closest to the position of the LOD peak. ^e^ This QTL on LG5 might be equivalent to the QTL by Battilana et al. [15] and Duchêne et al. [16]. ^f^ This QTL on LG2 might be equivalent to the QTL by Battilana et al. [15] and Duchêne et al. [16].**

## Table S6. Newly designed primers used in this study

| **Marker name** | **Linkage group (LG)** | **Physical position^a^** | **Forward primer** | **Reverse primer** | **Size^b^** | **Motif** |
| --- | --- | --- | --- | --- | --- | --- |
| Nifts5-50910 | 5 | 3,838,588 | GCAAAGCATAAGAGCCTTGATT | TGGTTGTCATTTTGATCACTCC | 232 | TC |
| Nifts5-50937 | 5 | 3,990,633 | AACCAATGTTTAGGCCAGTTTC | GCTCCCATCATTTCTCTTTTGT | 197 | TA |
| Nifts10-3830 | 10 | 794,040 | TGGAAAGAACCATAAAGCACAA | CACCCTTGATCTTCACTCTCCT | 175 | AT |
| Nifts10-3846 | 10 | 872,744 | CTTCTGTTATGGCAAAGATCCTG | GTTTCTTGTGGCCTCAGTATTGTGAGTTTC | 174 | TC |
| Nifts10-3863 | 10 | 974,842 | TTGCTGAGATCTCTTCCTTTGAG | CCCTGACTGGTCAAAGATTTACA | 249 | CAT |
| Nifts10-3911 | 10 | 1,232,427 | CTTAATGGCATGTTCCTTGTCC | AGGACGTACCTGTCAGGAAAAA | 208 | TTA |
| Nifts10-3928 | 10 | 1,293,018 | GCAAGTGCAATTTCTTTAACACC | TTACCATCCTGCAAAAAGAAAAA | 201 | CA |
| Nifts10-3935 | 10 | 1,317,653 | ACTTTTGCTCCTTCGTTTTTCTT | AGCCAATACACCAATCAATTCAC | 188 | TCT |
| Nifts10-3936 | 10 | 1,321,439 | TCAAACCGTTAAAGCTGCTAATC | TTTTGTCCTTTGGACACTGCTAT | 243 | AG |
| Nifts10-3938 | 10 | 1,339,984 | GCACGTTAAAGGAAAATGTTGTT | TTAAGTGGGGGAAATTAAAGAGG | 218 | GA |
| Nifts10-3940 | 10 | 1,343,749 | CATAGGTTGATTCTTTGATGTCCA | CTGCACTCGTCTGCTAGTCTTG | 191 | AT |
| Nifts10-3949 | 10 | 1,430,730 | CGGAGTCGTTTTGATCTGTAATC | GTGTGGAAGTTGAATTGTGGAAT | 210 | ATATC |
| Nifts11-7343 | 11 | 5,018,964 | TCGAAACAATTCTCAAACCAAAC | ATATACCCATATGCCCATTGACC | 160 | AT |
| Nifts11-7425 | 11 | 5,476,845 | TTGGATGAGAACCCTATGATTTG | GTCTGCAGAACAGTGGAAAGATT | 223 | TC |
| Nifts11-7518 | 11 | 6,077,520 | GAATACTAACGGTGAAATTTAAAATCG | GACCCGTAAATTTTGGTCATGT | 186 | ATA |
| **^a^ Starting position of each SSR in the 12X reference genome. ^b^ Expected sizes of PCR products based on the reference genome sequence. ^c^ Two different primer sets were developed for the same locus; genotyping results of Pop AC individuals were identical with both sets, with fragment size differences owing to the different primer positions.** | | | | | | |
